# Molecular Dynamics Simulations and Electric Field Poling of Covalently Bonded Chromophores at Poly(methyl Methacrylate)

**DOI:** 10.1021/acs.jpcb.6c01187

**Published:** 2026-06-15

**Authors:** Nils M. Denda, Oguzhan Albayrak, Henning Menzel, Carolin König, Peter Behrens, Andreas M. Schneider

**Affiliations:** † Cluster of Excellence PhoenixD (Photonics, Optics, and Engineering − Innovation Across Disciplines), 30167 Hannover, Germany; ‡ Institute of Inorganic Chemistry, 26555 Leibniz University Hannover, 30167 Hannover, Germany; § Institute of Technical Chemistry, Technische Universität Braunschweig, 38106 Braunschweig, Germany; ∥ Institute of Physical Chemistry and Electrochemistry, Leibniz University Hannover, 30167 Hannover, Germany

## Abstract

Embedding dipolar donor–acceptor molecules in polymer matrices is a promising route to novel optical materials. The noncentrosymmetric alignment of these chromophores is crucial for nonlinear optical activity in the application case. The present study proposes a novel simulation protocol, specifically designed to investigate the relaxation of chromophore alignment within a polymer matrix. This study contrasts two synthesis approaches: the first involves simple doped host–guest materials, and the second involves covalently bonded chromophores to the side chain of the host polymer. Covalent integration of chromophores results in higher glass transition values, thereby enhancing long-term alignment stability. The alignment stability of noncovalently bonded chromophore systems starts to decrease dramatically at 30 to 50 K above the observed glass transition. Covalently bonded chromophores show an enhanced alignment stability for the same temperature range above the observed glass transition. However, chromophore orientation is more constrained for chromophores that are covalently bonded to the polymer host compared to simple doped chromophore polymer systems. This study underscores the merits and limitations of covalent chromophore incorporation, delineating a paradigm for the exploration and development of novel, promising hybrid materials through simulation methods.

## Graphical abstract

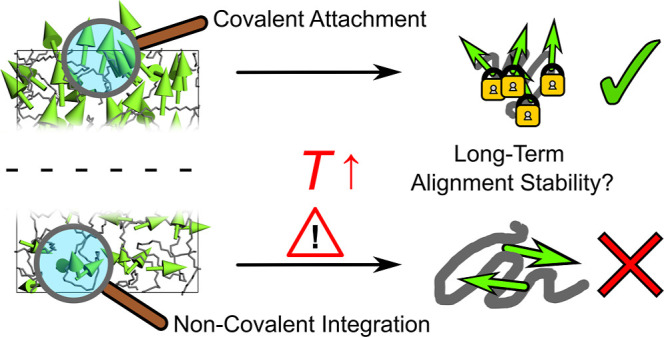

## Introduction

Electro-optic (EO) active materials are urgently required for future telecommunication applications, e.g., broadband communication (MHz to THz).^1^ Other applications include detection, sensing, imaging, metrology, spectroscopy, and super or quantum computing.^1–3^ Examples of photonic applications involve fiber optics, waveguide devices, interconnects, couplers, gratings, switches, and filters.^1–3^

Novel polymer-based materials are replacing traditional inorganic, crystalline materials, e.g., LiNbO_3_, due to their advantageous properties for applications, including bandwidth, dielectric constant, half-wave voltage, EO coefficients, and mechanical flexibility.^3^ In addition, polymer-based hybrid materials feature a less complex but more versatile processability (e.g., solution processability).^2,4^ This results in reduced material cost and simple property tuning.^4^ Ultimately, vertical integration on an all-polymer optical chip becomes possible.^2^

Polymer-based host–guest materials comprising organic chromophore molecules doped as a guest are a promising class of materials for future nonlinear optical (NLO) applications. For this purpose, chromophore molecules are specifically designed to exhibit a large first hyperpolarizability β. The simplest case of such a chromophore consists of an electron donor group and an electron acceptor group, connected via a π-electron system. This structural configuration facilitates the rapid movement of electrons across the molecule. Applying a weak electric field to chromophore-doped polymer materials may result in optical activity when all chromophores are in a noncentrosymmetric alignment.

This alignment within the polymer matrix may be obtained by the electric field poling process (above the glass transition *T*_g_) to render the material EO active. After poling and cooling to room temperature, the vitrified state of the polymer keeps the chromophores aligned. Subsequent to this material activation, NLO effects, e.g., the Pockels effect, can be utilized in various applications, provided that chromophores are maintained in an aligned state within the vitrified polymer host.

However, polymer-based NLO materials continue to face challenges that necessitate further investigation through experiments and simulation studies. The poling efficiency (i.e., the dipole orientation in the electric field) and long-term stability of chromophore alignment are especially crucial for developing materials that can be applied successfully and sustainably.^2,3^

In order to achieve a large, long-lasting EO effect, the polymer matrix must contain high concentrations of well-aligned chromophores.^5^ However, materials that are merely doped with chromophores that are not covalently bonded to the host may undergo aggregation, phase separation, and even crystallization at a certain concentration, leading to a deteriorated EO effect.^3,5–10^ Several synthetic strategies for chromophore design have been proposed as a means of reducing aggregation. One approach involves the utilization of bulky substituents. However, this strategy does not completely solve the aggregation problem.^10^ Other approaches comprise chromophore shape engineering, site isolation of chromophores, and multichromophore dendrimers, requiring well-elaborated synthesis conditions.^11^ Another concern with merely doped chromophore host–guest materials is the long-term alignment stability. It is well-known that alignment stability depends on glass transition, which is often lowered in highly doped noncovalent host–guest systems.^5^

Current approaches consider chromophores covalently bonded as side chain or directly integrated into the main chain (backbone) of the polymer host.^3,7,12^ The synthetic routes are often straightforward. Covalent integration can be carried out either during the polymerization step as copolymerization or after polymerization by applying, e.g., “click chemistry” to covalently bond the chromophore as side group.^3,7,12^ The covalent bonding of chromophores to polymer hosts is a promising approach, as it allows for the incorporation of high concentrations without the undesirable effects of aggregation or phase separation.^3,7,12^ Furthermore, the glass transition is not lowered due to the absence of a plasticizing effect, which presumably increases long-term alignment stability.^12^

In this context, simulation studies are an appropriate method for gaining insights at the atomistic level. These studies reveal structure–property relationships and promote material development. The first fully atomistic molecular dynamics (MD) simulations of guest doped (noncovalent) hosts were conducted by Kim and Hayden.^13^ A few more studies followed, investigating the effects of different chromophore shapes,^14^ the concentration dependence of chromophores,^6^ or macroscopic NLO activities.^15–17^ Recently, the first fully atomistic MD simulations of covalently bonded chromophores to the side chain of poly­(methyl methacrylate) (PMMA) were published.^18,19^ In these latest simulation studies, the glass transition and the poling efficiency (i.e., the dipole orientation) at comparably low poling field strengths (for simulations, i.e., 0.3 kV μm^–1^ to 1 kV μm^–1^) were investigated. Additionally, various types of inter- and intramolecular interactions were identified. However, a comparison of the results of the aforementioned simulation studies with experimental *T*_g_ investigations was not conducted, nor was the relaxation behavior of the chromophores examined. The question remains, whether the chromophores are fully equilibrated after 100 ns simulated poling at these comparably low poling fields of 0.3 kV μm^–1^ to 1 kV μm^–1^ for the time scale of simulations.

The objective of the present paper is to complement and extend the previous simulation studies. We provide a comprehensive simulation protocol for the investigation of covalently bonded chromophores: (a) We explore the glass transition *T*_g_ shift from noncovalent incorporated chromophores to side chain bonded chromophores in experiments and simulation studies (b) We investigate the optimum poling efficiency (i.e., the dipole orientation at elevated poling fields *E* = 5 kV μm^–1^). and (c) demonstrate the (long-term) alignment stability in direct comparison to noncovalent systems. (d) The EO activity is estimated in terms of the EO coefficient *r*_33_. (e) Finally, we propose an approach for the fast (computational efficient) exploration of novel chromophores or novel host–guest compositions, thereby revealing promising new acceptor groups.

A comparison of the chromophore candidates from our previous study^17^ and the current study is presented in Figure 1. Chromophore C3 contains a tricyanopyrroline (TCP) acceptor group, whereas chromophore (MA)­B1 is based on a tricyanofuran (TCF) acceptor, which has also been known for a long time.^20^ Over the past two decades, the TCF group was extensively employed in experimental^7,21–24^ and simulation studies.^8,12,19,25–31^ It is widely regarded as one of the most powerful and popular acceptor groups.^3,4,32^ We have employed the TCF acceptor because of its superior chemical stability compared to the TCP acceptor.^3^ The chemical stability includes various harsh reaction conditions, e.g., elevated temperatures during cross-linking, or the presence of radicals during copolymerization.^3,7,21,33^

**Figure 1:**
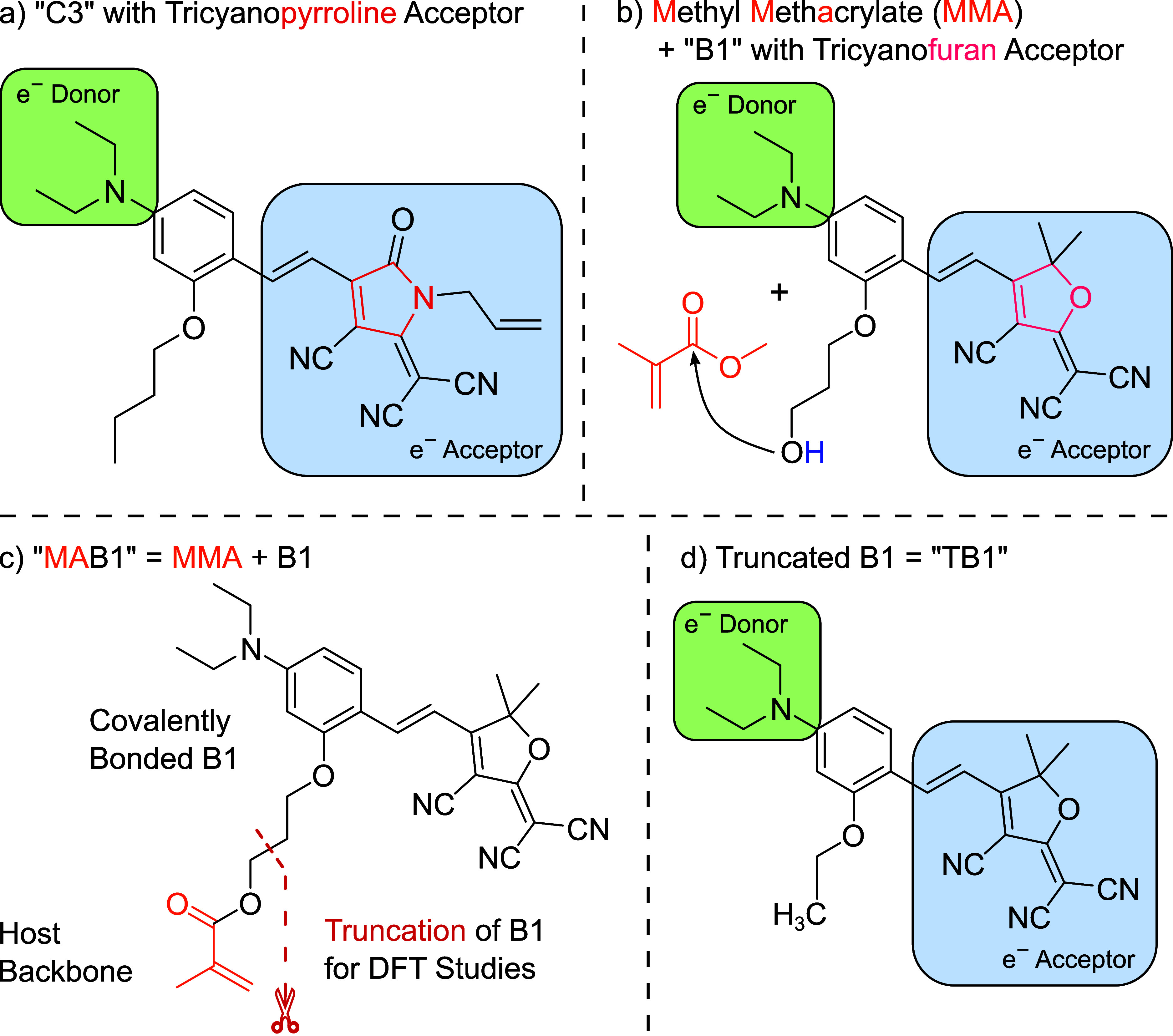
Chromophore “C3” (a) from our previous study^17^ in comparison to chromophore “B1” (b), that is covalently bonded to methyl methacrylate (MMA + B1 = MAB1) before polymerization (c). For density functional theory (DFT) studies and the calculation of the electro-optic coefficient the optically active part of the chromophore is cut out from the host backbone and saturated with a hydrogen atom, resulting in “truncated B1 = TB1” (d).

## Computational Methods and Experimental Details

Force field (FF) simulations were performed in BIOVIA Materials Studio 23.1 ^34^ employing the COMPASS III force field.^35^ Energy minimization (EM), molecular dynamics (MD) simulations and electric field poling were performed using the Forcite module. Chromophore energy minimization, density functional theory (DFT) calculations and coupled-perturbed Hartree–Fock (CPHF) calculations were carried out in Gaussian 16, Revision B.01.^36^

### Chromophore Characterization

The B1 and truncated B1 (TB1) chromophore structures were energy minimized employing FF methods. Afterward, the chromophore structures were further optimized using DFT methods (B3LYP^37,38^ /aug-cc-pVTZ^39–41^ ). The electrostatic potential (ESP) fitting method was applied to obtain atomic charges via the Merz–Kollman^42,43^ method. Default COMPASS III FF atomic charge parameters result in a significantly lower dipole moment of the chromophore than charge parameters calculated with DFT methods, as is well-known from previous molecular modeling studies.^6,13,15,16,25^ In order to simulate proper behavior of the chromophore in the applied electric field, atomic charges from DFT calculations were employed.

Time-dependent density functional calculations were carried out with the CAM-B3LYP^44^/aug-cc-pVTZ method in Gaussian to obtain excitation energies, as recommended for large organic chromophore molecules in ref 45. These calculations were applied on the DFT optimized structures with a solvent reaction field (PCM, polarizable continuum model)^46–48^ to account for effects arising from the polymer environment. Diethylamine (ε = 3.5766) was chosen as solvent because its permittivity is close to the low frequency permittivity of poly­(methyl methacrylate) (ε = 3.6).^49^ All permittivity values in this paper are expressed in relation to the vacuum permittivity, thus being presented without units.

Polarizability and hyperpolarizability values were obtained via CPHF^50–52^ calculations on DFT level with CAM-B3LYP/aug-cc-pVTZ. The polarizability and hyperpolarizability values were also calculated in a solvent reaction field as described above.

### Model Development and Simulation Protocol

Polymer related properties, e.g., the glass transition, are strongly dependent on the model size. Two different model sizes were created for each purpose to meet a good trade-off between computational effort and good representation. The larger-scaled models (around 20,000 atoms) were generated for the glass transition analysis. The smaller models (approximately 5000 atoms) were created to explore larger time scales and to ensure proper relaxation, e.g., chromophore alignment in the host polymer. The small models offer the advantage of expedited and concurrent mass production of MD trajectories. It was estimated that these models are sufficiently large to provide reliable statistics on chromophore alignment and relaxation processes.

In our previous modeling study, we identified 5000 atoms as the minimum threshold for host–guest modeling. This ensures that low concentration host–guest models contain more than five chromophore molecules in the unit cell. Small models are more tractable, especially for large time domain modeling. However, creating a set of independent models is recommended to provide a well-balanced (representative) dipole orientation statistic by parallel computation of independent models. In our previous study, we created a set of nine independent models. The present study has a model set size of five because creating the models is more elaborate, and the number of included chromophores is larger than in our previous study. A comparison of the model compositions from the previous study and the current study is presented in Table 1. Note that the estimation of 5000 atoms is based on our model systems. This estimation depends on the number of atoms or the mass of the chromophore in relation to the repeat unit/polymer size (in units of atoms or total weight fraction).

**Table 1:** Host–Guest Model Compositions (MMA = Repeat Units Without Chromophore Side Group, Chr = Chromophore)^a^

Label of Model Set^b^	Model Size^c^	#Chains	#MMA /Chain	#Chr	*M*(Chain) /kg mol^–1^	#Atoms
2 mol % C3	s	3	100	7	10.0	4947
4 mol %	s	3	100	12	10.0	5262
5 mol %	s	3	100	16	10.0	5514
7 mol %	s	3	100	22	10.0	5892
9 mol %	s	3	100	28	10.0	6270
2 mol % C3	*l*	14	100	34	10.0	23170
5 mol %	*l*	14	100	77	10.0	25879
9 mol %	*l*	14	100	132	10.0	29344
6 mol % B1	s	3	100	18	10.0	5586
8 mol %	s	3	100	26	10.0	6066
17 mol %	s	3	100	60	10.0	8106
6 mol % B1	*l*	9	100	54	10.0	16758
8 mol %	*l*	9	100	78	10.0	18198
17 mol %	*l*	9	100	180	10.0	24318
6 mol % MAB1	s	3	94	18	12.4	5478
8 mol %	s	3	104	27	14.9	6549
17 mol %	s	5	49	50	9.9	7135
6 mol % MAB1	*l*	9	94	54	12.4	16434
8 mol %	*l*	9	104	81	14.9	19647
17 mol %	*l*	15	49	150	9.9	21405

a The model compositions of C3 host–guest systems are taken from ref 17 and are presented for comparison, Ⓒ 2025 The Authors.

b Small C3 host–guest model sets contain nine independent models, large C3, B1, and MAB1 model sets contain five independent models.

c Small models (s) are used for poling and relaxation simulations, whereas large models (*l*) are used for glass transition analysis simulations.

In our previous study, we found that poly­(methyl methacrylate) models containing less than 5000 atoms were too small to reliably model the glass transition. Generally, polymers with larger repeat units probably require more total atoms (respectively more than 300 repeat units). In our earlier study, we created polymer chains with 100 repeat units for practical reasons, as reported previously.^13,15,17^ In contrast to prior studies,^13,15^ we increased the number of polymer chains (respectively repeat units) to more accurately model the glass transition.^17^

In this study, we employ our previously developed simulation protocol.^17^ For clarity, the essential settings and simulation steps are summarized in Figure 2. For the purpose of this study, the protocol was adapted to include covalently incorporated chromophores (step 1, Figure 2) and the relaxation protocol was extended to include an additional high-temperature simulation at 500 K (5s.b.iii, Figure 2). Pure polymer chains (for simple host–guest models) and polymer chains with covalently incorporated chromophores were constructed with the “Random Copolymer Builder” in Materials Studio (step 1, Figure 2). The methyl methacrylate repeat unit was assigned with default COMPASS III charges, whereas the chromophore repeat unit was equipped with DFT calculated charges. This procedure is common for modeling of highly functionalized chromophores in polymer matrices,^6,25^ since default atomistic force field charge parameters are designed to cover a broad range of different materials. However, chromophores are specially designed to exhibit a well-pronounced and characteristic charge distribution. The different chain compositions relied on preliminary experimental analyses and are documented in Table 1.

**Figure 2:**
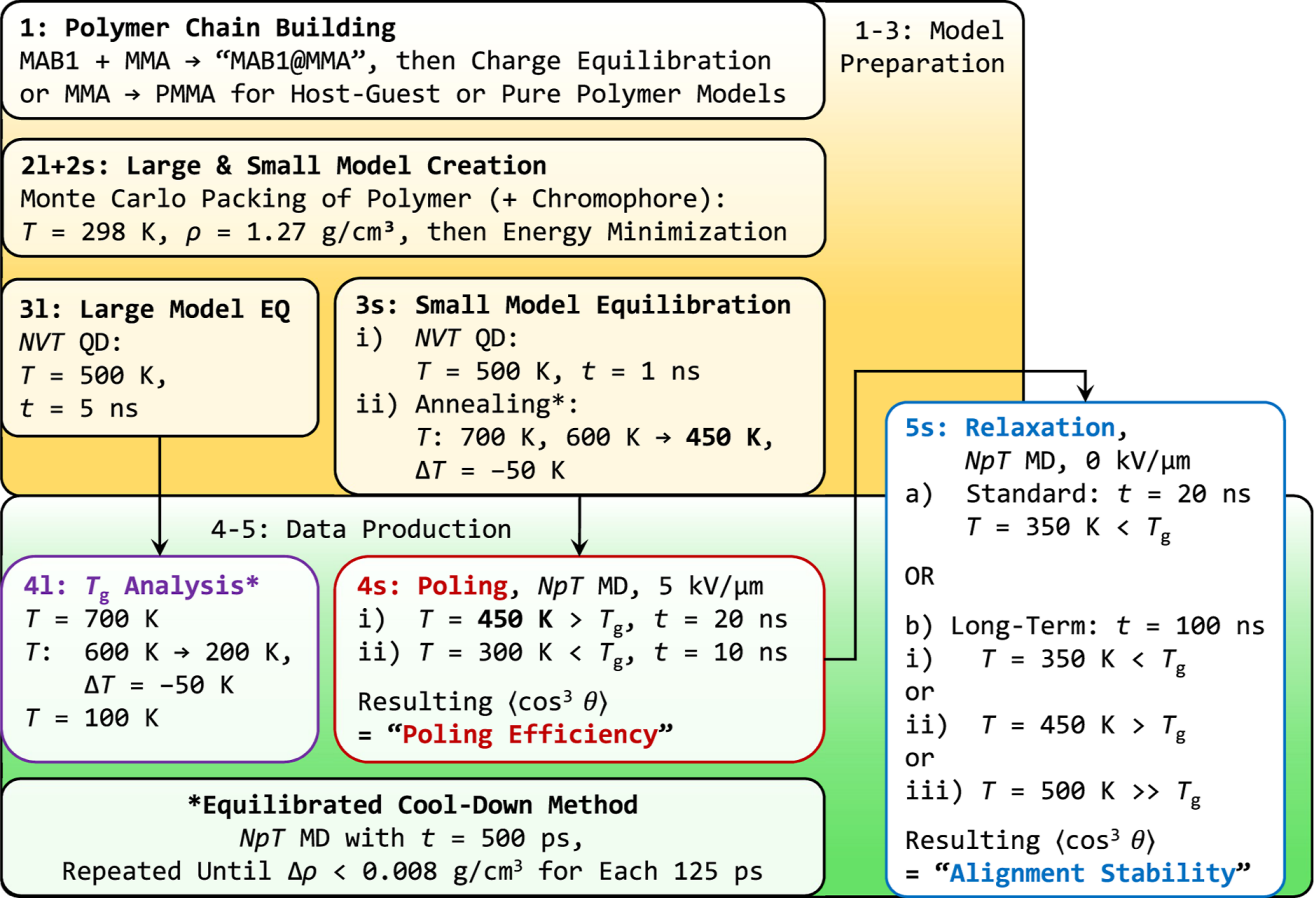
Flowchart of our simulation protocol. EQ: equilibration; (P)­MMA: poly­(methyl methacrylate); *l*/s: applied to large/small models; QD: quench dynamics simulation; ρ: density; *T*_g_: glass transition; *t*: simulation time; ⟨cos^3^θ⟩: order parameter.

The model compositions of the C3 host–guest systems are taken from ref 17 and are presented for comparison.

After polymer chain building, the charges were equilibrated with the “Edit Charges” dialogue. Atomic charge variation occurred at the third decimal place, and showed no significant effect on the dipole moments of the repeat units (<0.4 D per repeat unit). However, this preparation step resulted in an overall neutral charge for the entire chain. Host–guest models were created using the frequently used^6,13–17,53–55^ Amorphous Cell module in Materials Studio. The target temperature and density were chosen to be 298 K and 1.27 g cm^–3^, which is slightly higher than the experimental density (1.188 g cm^–3^)^49^ in order to obtain a dense host–guest structure (compare refs 15 and 17 ).

Five independent models were constructed for each mass concentration of the chromophore systems B1 and MAB1. An energy minimization was performed after the Monte Carlo packing (simulation protocol step: 2*l* + 2s, Figure 2). Subsequently, a quench dynamics (QD) simulation was carried out. This is an MD simulation with additional energy minimizations after specific time intervals, in order to obtain a collection of low energy structures. The QD was conducted in the *NVT* ensemble (isochoric–isothermal conditions) using a Nosé thermostat^56^ for 5 ns at 500 K for large models (step: 3*l*, Figure 2) and for 1 ns at the same temperature for small models (step: 3s.i, Figure 2). 100 energy minimized frames were obtained in each simulation and the frame with the lowest energy was taken as initial structure for further simulation steps. For small models, an annealing step was conducted (step: 3s.ii, Figure 2), after QD simulation and before poling to equilibrate the models at the poling temperature of 450 K. For large models, glass transition analyses were carried out in the temperature range from 700 to 100 K (step: 4*l*, Figure 2).

As in our previously developed poling and relaxation procedure, the electric field was applied in the *z*-direction in a *NpT* ensemble with a Nosé thermostat and Berendsen^57^ pressure control. The poling includes two steps: first (step: 4s.i, Figure 2), chromophore alignment is facilitated at elevated temperatures above the glass transition temperature. Subsequently (step: 4s.ii, Figure 2), the chromophore alignment is conserved in the vitrified polymer host, below the glass transition temperature. Finally, the relaxation step (5s, Figure 2) is introduced to trigger reorientation and investigate the (long-term) stability of the chromophore alignment. As with our previously developed MD approach,^17^ the order parameter ⟨cos^3^θ⟩ was calculated frame by frame and averaged over every 2 ns. The terms “poling efficiency” and “alignment stability” were established, referring to the average order parameter value ⟨cos^3^θ⟩ after poling (step: 4s, Figure 2) or relaxation (step: 5s, Figure 2), respectively.

In our previous study, different electric field strengths were applied ranging from 0.5 kV μm^–1^ to 5 kV μm^–1^. The field strength of 5 kV μm^–1^ was chosen for the majority of simulations,^17^ although it is much stronger than that applied in experimental setups (0.1 kV μm^–1^ to 0.2 kV μm^–1^).^10,58^ However, it is necessary to achieve equilibration on the time scale of simulations. This dilemma is commonly known for molecular modeling and is examined and discussed in more detail in our previous paper.^17^ In the present study, we also applied an electric field strength of 5 kV μm^–1^ for all poling simulations, because we are interested in the optimum poling efficiency. The concept of “optimum poling efficiency” is defined as the observed order parameter value resulting from optimal alignment of the chromophores’ dipole moments, which in turn produces a modeling reference state. From an experimental perspective, the reference state that is achieved at the end of the poling stage (4s) as illustrated in Figure 2 can be regarded as a material preparation step. It is important to emphasize that the field strength employed in this context should not be equated with the electric field in the electro-optical application scenario. The latter is related to electron polarization across the donor–acceptor chromophore, a subject that extends beyond the scope of the molecular dynamics simulations presented herein. The present molecular dynamics study focuses on the dipolar (long-term) relaxation behavior of the chromophores in the absence of an electrical field. The poling efficiency may depend on the model type (covalently bonded chromophores or simple doped systems), the model composition (type and composition of chromophores and polymer, e.g., the type and number of included atoms, steric hindrance, etc.), poling conditions (e.g., electric field strength, temperature, simulation duration), and on the single model. Therefore, we have elaborated a set of modeling parameters (electric field strength, temperature, simulation duration, number of atoms in one model, model set size), that are introduced in the computational methods section and discussed at the beginning of the results and discussion section (Order Parameter Analysis, Poling, and Relaxation Behavior, see Figure 4). This discussion precedes the novel analysis of (long-term) relaxation behavior.

### Order Parameter and Optical Activity Estimation

The alignment of a set of dipoles is described by the order parameter ⟨cos^3^θ⟩ and is required to estimate the extent of electro-optical activity.^59^ The angle θ is introduced to describe the orientation of one dipole in the applied electric field.

Theoretical models have been developed to calculate the order parameter, e.g., of an ideal gas consisting of rigid, noninteracting spheres. The third Langevin function

1 $$\left\langle \cos^{3}\theta \right\rangle =L_{3}\left(x\right)=\left(1+\frac{6}{x^{2}}\right)\coth x-\frac{3}{x}\left(1+\frac{2}{x^{2}}\right)$$

with

2 $$x=\frac{\mu E}{kT}$$

where μ is the molecular dipole moment, *E* the applied electric field, *k* Boltzmann’s constant and *T* the absolute temperature,^5,13^ can be used to calculate the order parameter.

The electro-optic (EO) tensor element *r*_333_ is used to describe macroscopic optical activity.^58^ Typically, the first two indices are contracted for reasons of symmetry (*r*_*ijk*_ = *r*_*jk*_ = *r*_*ik*_), resulting in *r*_333_ = *r*_33_.^58^ The EO coefficient *r*_33_ is related to the second-order susceptibility *χ*_*zzz*_^(2)^, and the molecular hyperpolarizability β by^58,60^

3 $$r_{33}=\frac{2\chi_{zzz}^{\left(2\right)}}{n_{z}^{4}}=\frac{2N_{c}\beta f_{0}f_{\lambda }^{2}\left\langle \cos^{3}\theta \right\rangle }{n_{z}^{4}}$$

where *N*_c_ is the number density of chromophores, *f*_0_ and *f*_λ_ are local field factors, ⟨cos^3^θ⟩ is the order parameter of the chromophores, and *n*_*z*_ is the refractive index with respect to the polar axis. In the present study, we applied our previously developed polarizable continuum (PCM) approach^17^

4 $$r_{33}=\frac{2N_{c}\beta^{PCM}\left\langle \cos^{3}\theta \right\rangle }{{\left(n_{z}^{PCM}\right)}^{4}}$$

for an implicit treatment of local field effects and a straightforward calculation of the EO tensor element. The full derivation and all necessary equations to calculate the refractive index *n*_*z*_^PCM^ are provided in the Supporting Information of our previous paper.^17^

### Sample Preparation and Compositions from the Experiment

The chromophore B1 synthesis was adopted from ref 28. After the chromophore B1 was coupled with the methyl methacrylate (MMA) moiety (B1 + MMA → MAB1), it was copolymerized with additional MMA via free radical polymerization. Different feed ratios of MAB1 (methyl methacrylate containing chromophore) and MMA (methyl methacrylate) were dissolved and degassed in dimethyl sulfoxide and polymerized with azobis­(isobutyronitrile) at 65 °C for 3 days to 7 days.

$$nMAB1+mMMA\overset{AIBN}{\underset{DMSO}{\to }}{\left[MAB1\right]}_{n}-{\left[MMA\right]}_{m}=\;\prime \prime MAB1@MMA\prime \prime$$

The product was precipitated in cold diethyl ether.

The copolymers were purified via the repeated precipitation of the crude product using tetrahydrofuran (THF, dissolving solvent) and diethyl ether (nondissolving solvent) to remove unreacted B1 or MAB1 from the MAB1@MMA copolymer. Purified and thin-layer chromatography validated polymers were measured with gel permeation chromatography (GPC). Molecular weight distributions of the polymers were measured on a “PSS SECcurity^2^” instrument (Polymer Standards Service (PSS), Mainz, Germany) equipped with “PSS SDV” columns (precolumn 5 μm, 8 × 50 mm and two main columns: 10 μm, 1000 Å, 8 × 300 mm and 10 μm, 1000 Å, 8 × 300 mm), and a refractive index detector. The samples were dissolved in THF and passed through the columns at a flow rate of 1 mL min^–1^ at 40 °C. The system was calibrated with narrow polystyrene calibration standards.

In addition to the MAB1@MMA covalently bonded chromophore samples, three different noncovalent (doped) chromophore host–guest samples (a-c) of B1 and PMMA were prepared (“B1 in PMMA”). The B1 chromophore (samples (a-c): 10 mg) and 15 kDa PMMA^61^ (a: 5.433 g, b: 4.075 g, c: 1.918 g) were dissolved in THF pairwise, respectively, and left stirred overnight. Dissolved polymer–chromophore solutions were poured into a drying dish and left to dry for 6 h at 50 °C in the vacuum oven.

In polymer incorporated chromophore contents and chain masses are presented in Table 2. The mole percent content of incorporated chromophores was determined by a NMR analysis. The NMR analysis of the polymers was carried out with a “Bruker Avance II 300” (Bruker BioSpin, Ettlingen, Germany). The chromophore concentration in the polymers was calculated via comparison of the chromophore proton integrals with respect to the integral of the methoxy protons of the polymer backbone.

**Table 2:** Overview of the Experimental Samples

sample	mol % Chr	*M*_n_/kg mol^–1^	*M*_w_/kg mol^–1^
B1 in PMMA^a^	6.0	–	15.0
	7.8	–	15.0
	15.3	–	15.0
MAB1@PMMA	6.3	7.8	15.1
	12.5	4.1	8.0
	16.7	9.0	18.6
	20.0	7.6	15.9

a Molecular masses are taken from the product data sheet.^61^

### Glass Transition Analyses of Experimental Samples

Thermal analysis of experimental polymer samples were measured with a “Netzsch DSC 300 Caliris” (Netzsch, Selb, Germany) differential scanning calorimetry (DSC) device. Measurements were performed in three cycles, each cycle ranging from 323 to 473 K with a heating and cooling rate of 10 K min^–1^. The first both temperature cycles are for the equilibration of the system and to remove the thermal history of the samples. The glass transition values of the inflection point of the third cooling cycle are presented herein. Glass transition calculations were carried out with the “Netzsch Proteus Software”.

## Results and Discussion

### Glass Transition Analysis

In Figure 3, all glass transition values from experimental samples (bullets) and simulation models (triangles) are summarized. Glass transition values of different chromophore systems (C3/B1/MAB1) and incorporation methods (covalent/noncovalent) over a large concentration range are provided. Measurements of materials with C3 incorporated are adapted from our previous paper,^17^ as a reference to the current study.

**Figure 3:**
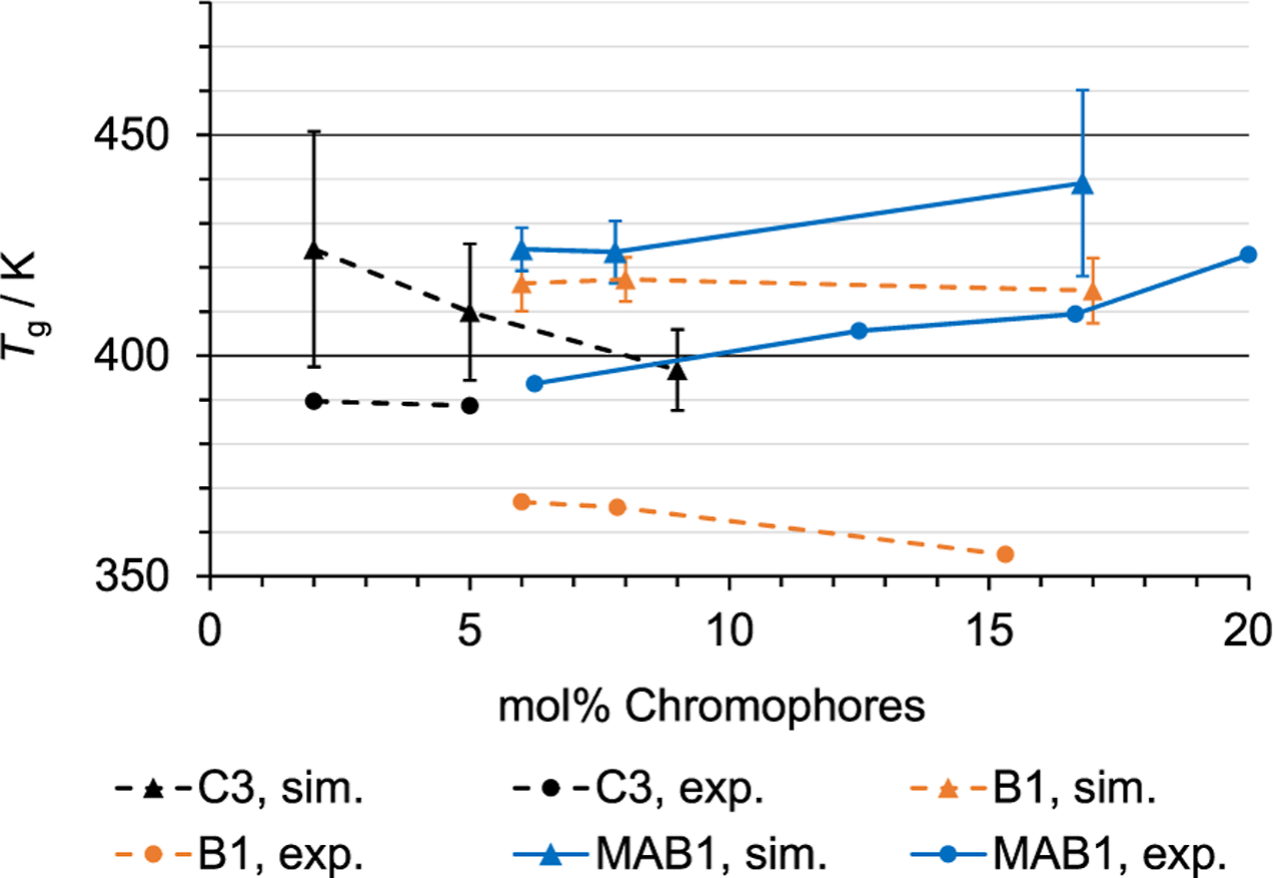
Glass transition *T*_g_ estimated from MD simulations (sim., triangles) in comparison to experimental values (exp., bullets) of host–guest models in dependence on the chromophore concentration. Lines are employed solely to guide the eye. Dashed lines: noncovalent host–guest models, solid lines: covalently bonded chromophores. *T*_g_ values of C3 in PMMA are adapted from ref 17 as reference, Ⓒ 2025 The Authors. Experimental values have uncertainties below 5 K, error bars are therefore omitted.

In general, the simulated *T*_g_ values are overestimated by 20 to 40 K compared to experimental measurements. This phenomenon is very common and has its origin in the large gap of cooling rates between simulation and experiment.^6,14–16,18,19,25^ Experimental results show a slight decrease of the *T*_g_ for noncovalently incorporated chromophores (C3 and B1). This decreasing trend is stronger pronounced for the C3 host–guest simulations, but not clearly visible for the much higher concentrated B1 host–guest simulation models. The B1 host–guest models show a rather constant *T*_g_ value, regardless of concentration. Still, the decreasing trend for *T*_g_ for noncovalently bonded materials is well-known and generally attributed to the plasticizing effect of host–guest doped materials.^5,6,14^

For covalently bonded chromophores an opposed trend is apparent, for both, the experimental measurements and simulation results. The increase in *T*_g_ for covalently bonded chromophores is expected, as it is well reported in literature.^5,19,62,63^ Apart from the shift of *T*_g_ values to higher temperatures for the simulation results, the trends for the incorporation method (covalent/noncovalent) and the dependence of chromophore concentration on glass transition are well corresponding between experiment and simulation.

### Order Parameter Analysis, Poling, and Relaxation Behavior

Figure 4 displays the poling efficiency and alignment stability of different host–guest simulations under standard poling and relaxation conditions (cf. Figure 2, step 5s.a). The novel simulation studies of covalently bonded MAB1 chromophores in PMMA are compared to noncovalently incorporated chromophores, B1 and C3 (adapted from our previous study),^17^ both in PMMA. Regardless of chromophore concentration, the noncovalently incorporated chromophores are easier to align in the polymer host, compared to the MAB1 chromophores (Δ⟨cos^3^θ⟩ > 5%). For the poling condition in this simulation study (5 kV μm^–1^, 450 K), both noncovalent model systems, C3 and B1, show roughly the same poling efficiency (around 90%). The order parameter decreases around 15% to 20%, resulting in a comparable alignment stability of 70% to 75% for both noncovalent chromophore model systems.

**Figure 4:**
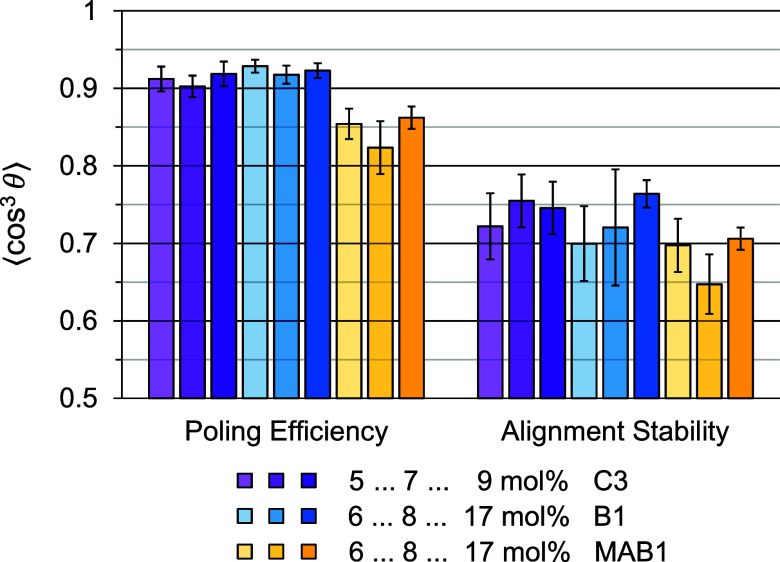
Poling efficiency and alignment stability of different chromophore systems with different concentrations in comparison. “C3 in PMMA” (purple, from ref 17, Ⓒ 2025 The Authors) as a reference. The average order parameter values ⟨cos^3^θ⟩ are plotted against the mole concentration of the respective model set. One model set is described by the average of nine independent (for C3) or five independent (for B1 and MAB1) structure model calculations. Error bars show respective standard deviations.

The poling efficiency value of the covalently bonded MAB1 model systems is 5% to 10% smaller, than for the noncovalent systems. The final alignment stability value is also slightly lower than in the noncovalent systems. However, the difference in alignment stability is only for the high concentration models of B1 and MAB1 significant. The decrease of the order parameter from poling to relaxation is for all models (noncovalent and covalent) approximately the same (−15% to −20%) in the standard relaxation protocol (20 ns, 350 K). The movability of the covalently bonded chromophore MAB1 is restricted to the polymer side chain, so that alignment and orientation processes are impeded, leading to a lower poling efficiency compared to the merely doped systems. The relaxation dynamics are discussed later in more detail.

Individual order parameter diagrams in the course of the poling and relaxation simulation for the covalently bonded chromophore systems were analyzed in detail and show a constant level of the order parameter after 10 ns for the standard poling and relaxation protocol (*E* = 5 kV μm^–1^; *T* = 450 K; for *t* = 20 ns), so that optimal alignment for the chosen poling conditions is reached. In addition to the standard poling conditions, stronger electric field strengths and higher temperatures were tested. All these diagrams are provided in the Supporting Information (section S1). Only a very strong electric field (10 kV μm^–1^, twice the standard poling field) significantly increased the poling efficiency (cf. Figure S1d). The alignment stability exhibits comparable behavior to that observed under standard poling and relaxation conditions. In conclusion, stronger electric fields and adjustments to the simulation protocol, e.g., higher temperatures, may be applied in simulations, especially when the apparent glass transition is elevated. However, in the present study, no remarkable differences were observed, so the previously developed poling and relaxation protocol is also applicable to the covalently incorporated chromophore systems, and the results are comparable to those of our previous studies. As we are interested in achieving optimal poling efficiency, we have not tested lower poling field strengths for the (MA)­B1 systems because equilibration is expected to take more computational effort (time), as previously noted.^17^

The previous paragraphs discussed the results of the standard poling and relaxation conditions of the whole model sets. Additionally, the effects of stronger electric field strengths, and higher temperatures were tested and discussed (Supporting Information section S1). Though slight differences were observed, the results of the different simulation models (covalently bonded chromophores and simple doped systems) are comparable under the elaborated simulation conditions, since, on average, the models reached the reference state of equilibrated optimal poling efficiency.

Figure 5 shows the order parameter ⟨cos^3^θ⟩ evolution for long-term relaxation (LTR) simulations (no electric field, for 100 ns). It is important to note that each diagram represents the evolution of a single model at the three specified temperatures. Furthermore, the relaxation curves were not normalized, allowing their values to be compared with the data in the other tables and figures. The order parameter values are included in the different diagrams, to facilitate the comparison of relative changes. The explicit poling efficiency (on the left) and the alignment stability (on the right) are dependent on the specific model system (covalently bonded, simple doped, and chromophore concentration). Therefore, a direct comparison of these single-model values across different diagrams is not appropriate. The following paragraphs discuss the relative changes in the order parameter.

**Figure 5:**
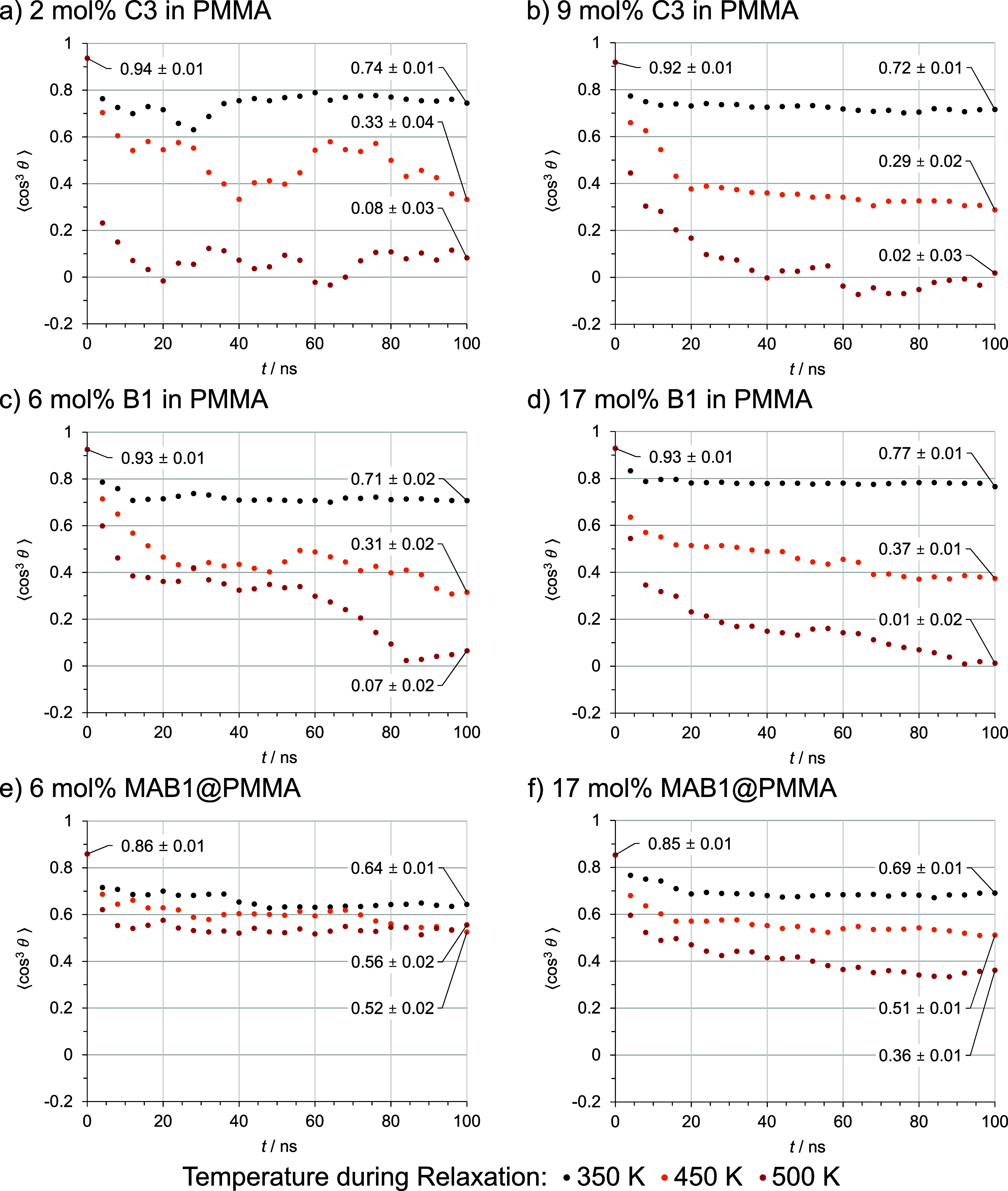
Order parameter ⟨cos^3^θ⟩ during long-term relaxation (LTR) simulations for different host–guest models. Figures (a,c,e) show low concentration models and Figures (b,d,f) show high concentration models of the corresponding model set. The LTR simulations of C3 in PMMA at 350 and 450 K are adapted from our previous study,^17^ Ⓒ 2025 The Authors.

The three specified temperatures for the LTR simulations are below *T*_g_ (350 K), above *T*_g_ (450 K), and far above *T*_g_ (500 K, cf. Figure 2, step 5s.b). The LTR simulations are carried out for all chromophore systems at the corresponding lowest and highest chromophore concentration. The LTR simulations of C3 in PMMA at 350 and 450 K are adapted from our previous study^17^ and are provided as a reference for noncovalent host–guest systems with lower chromophore concentrations. Our previously developed LTR simulation protocol^17^ was extended with a third simulation run at 500 K (cf. Figure 2, step 5s.b.iii), because of the higher *T*_g_ values of the covalently bonded chromophore systems.

All six models show a constant order parameter value at a temperature of 350 K (below all glass transitions), proving the alignment stability of every model system. At a temperature of 450 K, which is 30 K above the glass transition for B1 chromophore systems and low concentrated C3 systems, and 50 K above high concentrated C3 host–guest models, all these mentioned models show a dramatic order parameter decrease by more than 50%, to a value of around 30%. Covalently bonded chromophores in the MAB1@PMMA models have a glass transition in the range from 420 to 440 K, so these models benefit from the elevated glass transition and the covalent bond to the polymer host regarding the alignment stability. The covalently bonded chromophores do not have the same poling efficiency like the noncovalent systems (approximately 10% smaller), but they provide a better alignment stability. The order parameter value decreases by approximately 30% and results in an order parameter of around 52% (+20% compared to the noncovalent model systems). The relaxation behavior of C3 in PMMA and MAB1@PMMA at 450 K is illustrated by snapshots of the MD simulation in the Supporting Information (section S2, Figures S2 and S3).

For relaxation simulations at a temperature of 500 K, the apparent glass transition temperatures of the simulations are exceeded by approximately 60 K (MAB1 high conc.), 80 K (C3 and MAB1 low conc., and B1), and 100 K (C3 high conc.). Each model set exhibits a distinct relaxation behavior, as detailed below. Low concentrations of noncovalently incorporated chromophores (C3 low conc. model) cannot maintain alignment, the order parameter decreases to approximately zero. Higher concentrations of noncovalently incorporated chromophores (C3 high conc. or both B1 models) show a continuously delayed or stepwise decrease in the order parameter, ending at an average order parameter of approximately zero. Covalently bonded chromophore systems have been demonstrated to offer enhanced alignment stability, even at elevated temperatures. However, a decrease in alignment stability (by approximately 30% to 50%, to 56% (low conc. MAB1@PMMA) or 36% (high conc. MAB1@PMMA)) must be considered.

This implies that materials comprising noncovalently incorporated chromophores may exhibit resistance to short or occasionally temperature peaks that exceed their specified stability temperature. However, elevated temperatures frequently result in the complete inactivation of these merely doped materials. In contrast, covalently bonded chromophore materials are expected to demonstrate superior performance in analogous stress tests.

### Practical Considerations of Host–Guest Doped versus Covalently Bonded Chromophore Materials

Besides the disadvantage of a lower *T*_g_ in doped host–guest systems, one of the main advantages of these systems is that they are easier to prepare and allow for better control over chromophore concentration. In a doped system, the host material and chromophore are mixed in a solution at a specific ratio. After a homogeneous mixture is formed, it can be cast directly onto the substrates. However, in covalently bonded chromophore systems, the purification step is crucial after successful polymerization. The process of removing unreacted monomers or the catalyst used in the polymerization reaction is effortful. The purified polymer requires multiple precipitations, each of which causes an inevitable loss of product. Once purified, the polymer can be dissolved and cast onto a substrate. In doped systems, increasing the concentration of the host polymer requires more solvent for dissolution, affecting the properties of the casting solution. In covalently bonded chromophore systems, however, the chromophore concentration is fixed. Consequently, modifications to the concentration of the chromophore within covalently bonded chromophore systems require a new synthesis of the polymer with the desired chromophore-to-polymer repeat unit ratio.

### Chromophore Characterization and Modification

In Table 3, an overview of the optical properties of different chromophores is presented. The hyperpolarizabilities are presented for two cases: the values for the minimum wavelength (or maximum frequency) of the electro-optic (EO) experiment (λ­(EO) > λ_0_ + 300 nm), before notable absorption at λ_0_ begins; and the static case (with ν = 0 or λ → ∞), respectively. The former case represents the upper limit of expected EO activity under ideal conditions, while the latter case corresponds to the lower limit of expected EO activity.

**Table 3:** Chromophore Properties and DFT Calculated Absorption Wavelengths λ_0_ and Hyperpolarizabilities β Dependent on the Wavelength of the Electro-Optic Experiment^a^ λ­(EO)

chromophore	C3^b^	TB1	DNT	TCT	DNS
formula	C_27_H_29_N_5_O_2_	C_24_H_26_N_4_O_2_	C_22_H_26_N_4_O_6_	C_24_H_26_N_4_OS	C_22_H_26_N_4_O_5_S
*M*/g mol^–1^	455.6	402.5	442.5	418.6	458.5
λ_0_/nm	606	472	486	490	506
λ(EO)/nm	970	850	850	850	850
β_∥_/10^–30^ esu	641.2	283.8	356.4	403.1	607.2
Values for the Static Limit Below (ν → 0 or λ(EO) → ∞)					
β_∥_/10^–30^ esu	345.0	179.9	215.9	239.8	330.6

a The wavelength of the electro-optic experiment λ­(EO) is determined based on the wavelength λ_0_, i.e., the lowest absorption energy of the corresponding chromophore (λ­(EO) > λ_0_ + 300 nm).

b Properties of C3 are adapted from ref 17 as reference, Ⓒ 2025 The Authors.

For the straightforward estimation of the EO effect, the most important performance indicator is the total hyperpolarizability β_tot_, arising from the tensor element parallel to the dipole moment β_∥_ $\left(\beta_{tot}=\frac{5}{3}\beta_{∥}\right)$ .^64^ Therefore, the discussion of the first hyperpolarizability is limited to the tensor element parallel to the dipole moment β_∥_. The total hyperpolarizability β_tot_ and the polarizability values α are provided in the Supporting Information (section S3). The (hyper)­polarizability values of MAB1, B1, and TB1 are discussed in the Supporting Information (section S3). Figure 6 shows the previously developed C3 chromophore in comparison to B1, and TB1 of the current study, and in comparison to novel chromophores with modified acceptor groups (DNT, TCT, DNS), which are discussed in the next paragraphs.

**Figure 6:**
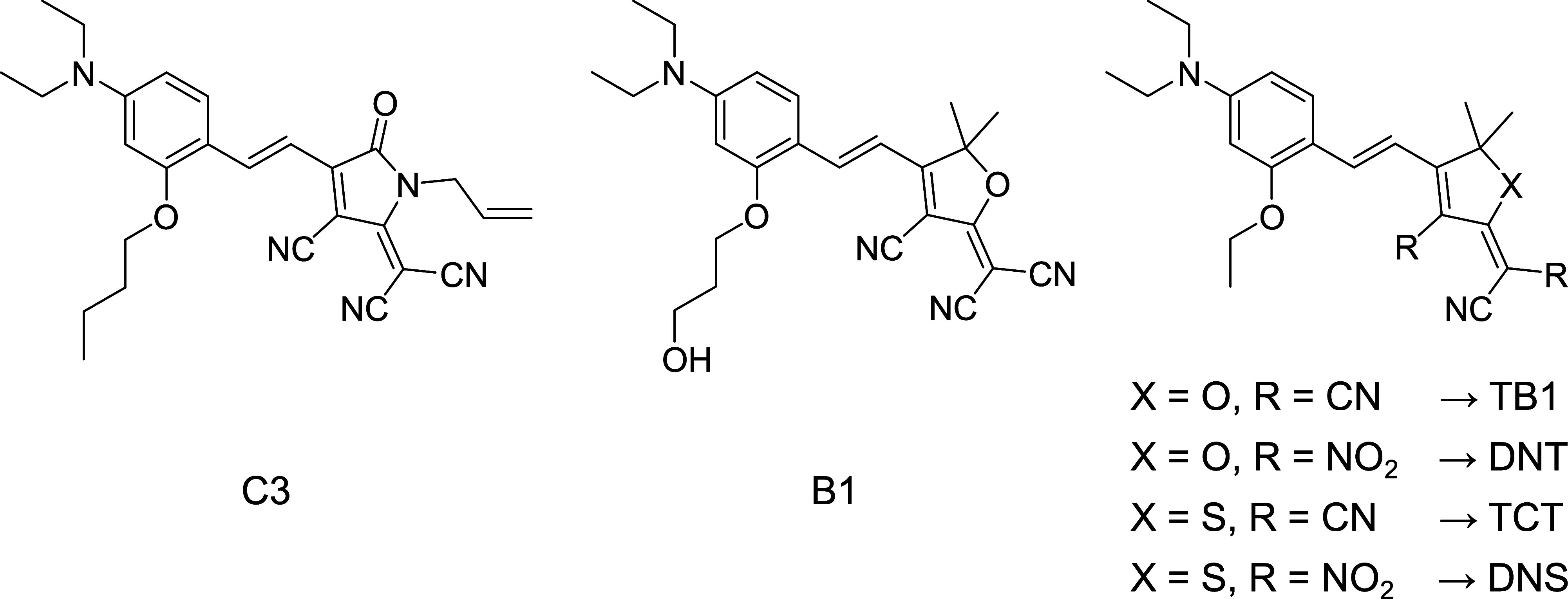
Direct comparison of the first set of chromophores (C3 and B1) and the acceptor group modifications on TB1 to DNT, TCT, and DNS. The tricyanofuran acceptor group has been altered, as seen in a patent.^65^ The acceptor group of DNS is a combination of the acceptor groups DNT and TCT. A synthesis route to this candidate was not found, yet.

In the static case, the hyperpolarizability of C3 is almost twice as high as for TB1 (Δβ_∥_ = 165 × 10^–30^ esu or Δβ_∥_ = 92%). At shorter wavelengths (vis/IR range) the difference in β_∥_ is even stronger pronounced and the value for C3 is more than twice as high as for TB1 (Δβ_∥_ = 357 × 10^–30^ esu or Δβ_∥_ = 126%). The most significant difference between C3 and TB1 is the electron acceptor group. Besides the “tricyano” structure element in both chromophores, the pyrroline acceptor (with oxygen and nitrogen) in C3 has a stronger electron withdrawing character compared to the furan acceptor in TB1 (only oxygen with electron withdrawing character).

As mentioned in the introduction, the tricyanofuran (TCF) acceptor was chosen because of its chemical stability. The hyperpolarizability value for TB1 is considerably lower than expected in comparison to C3. Based on these findings, we decided to modify the TCF acceptor group in our simulation studies in order to probe the hyperpolarizability values. The structural modifications are small (essentially electronic structure modifications) to avoid an impact on the results of the molecular dynamics simulations. This allows us to substitute the DFT-calculated properties of TB1 with those of DNT, TCT, or DNS in the calculation procedure for the EO tensor element *r*_33_ (presented in the next section). Other modifications are also possible for future chromophore developments, e.g., variation of the donor group or π-electron bridge, as commonly reported in the literature.^4,29,30,32^ For this study, however, our initial aim is to conduct small variations in the electronic structure. A more profound investigation of the acceptor group modifications to the ESP charge profile and sterical demand is included in section S4 of the Supporting Information.

The substitution of the cyano groups with nitro groups and the exchange of oxygen with sulfur leads to a slight red shift of the lowest absorption energy (Δλ ≈ + 15 nm) in both cases. The effect is approximately doubled (Δλ ≈ + 30 nm), if both changes are combined in the DNS acceptor group. The nitro functionalization enhances the hyperpolarizability by approximately 20%, the sulfur modification leads to an increase by approximately 33%, and combining the two modifications increases the hyperpolarizability by approximately 84% (all percentages refer to the static limit, for wavelengths below this limit, the increase is even larger).

To summarize this section, modifying the TCF acceptor of the TB1 chromophore revealed promising new chromophore candidates that have not yet been investigated in experiments. As a guide for future research, Quilty^9^ identified hyperpolarizabilities ranging from 800 × 10^–30^esu to 1000 × 10^–30^esu as promising for further experimental investigation. With regard to this statement, we have identified a new promising chromophore candidate with β_tot_(DNS) = 1012 × 10^–30^ esu, at λ = 850 nm, if the DNS chromophore is synthetically available. The synthesis of this chromophore has not yet been described in the literature.

This section outlines a straightforward and fast procedure for exploring new chromophore candidates. We propose cutting off the electro-optically active chromophore from the polymerizable unit (here: MMA) or any other large side groups to computationally efficiently calculate altered functionalities on the DFT level (without DFT calculations of large oligomers). This approach requires two precepts. First, the chromophore modifications predominantly affect the electronic structure, and second, the EO activity can be separated from the polymer host, as discussed in the Supporting Information (section S3). Otherwise, if large molecular fragments with sterical demand (significant higher molecular weight) are introduced, or highly polarizable side chains/polymerizable units of the polymer host are present, the entire simulation approach has to be revisited. This includes the MD simulations with regard to phase behavior investigations such as glass transition, poling, and relaxation (possibly with adjusted simulation conditions, e.g., temperature, electric field strength, simulation time, etc.), as well as order parameter analyses. Furthermore, if the polymer backbone or side group is expected to be in resonance with the charge transfer of the chromophore system, thereby affecting the optical properties of the covalently bonded chromophores, the polymer system should also be included in higher-level DFT calculations (e.g., oligomers of polymerized chromophores) to ensure the reliability of calculated EO activities. The next section will show, how the changed optical properties through the modified acceptor groups will manifest in the overall EO activity *r*_33_.

### Electro-Optical Activity Estimation

Table 4 lists the density of host–guest models ρ, the number density of chromophores *N*_c_, the order parameter ⟨cos^2^θ⟩ and ⟨cos^3^θ⟩ (after standard relaxation), the permittivity ε_λ_, the refractive index *n*_*z*_ and the EO activity *r*_33_ of MAB1@PMMA at the wavelength of the EO experiment λ­(EO). As reported in the previous section, the total hyperpolarizability of (T)­B1 is much lower compared to the previously investigated C3. Based on this result, modifications of the TCF acceptor group have been examined on DFT-level, resulting in three novel chromophores, namely DNT, TCT, and DNS. Since the modifications are small and mainly based on the fine-tuning of the electronic structure, it is assumed, that the phase behavior is largely unchanged, so that the order parameter values remain the same for the modified acceptor groups. Within this assumption, we have joined the DFT-calculated (hyper)­polarizabilities of the modified chromophores with the atomistic MD simulation results of MAB1@PMMA (order parameter, average densities), to calculate the altered EO responses. An evaluation of the ESP charge profiles and steric profiles of the studied chromophore candidates can be found in section S4 of the Supporting Information. Additionally, section S5 of the Supporting Information explores alternative solvents for the PCM calculations and their impact on the electro-optic activity.

**Table 4:** Summary of Data for MAB1@PMMA Modeling (Values After Relaxation)^a^

Model Set	ρ /g cm^–3^	*N*_c_ /10^20^ cm^–3^	⟨cos^2^θ⟩	⟨cos^3^θ⟩	ε_λ_	*n*_*z*_	*r*_33_ /pm V^–1^
PP^b^	1.12	–	–	–	2.19	1.48	–
6 mol %	1.11	3.22	0.77 ± 0.03	0.70 ± 0.03	2.30	1.57	14.5 ± 0.9
8 mol %	1.12	4.07	0.73 ± 0.03	0.65 ± 0.04	2.34	1.60	16.1 ± 1.2
17 mol %	1.13	6.84	0.78 ± 0.01	0.71 ± 0.01	2.46	1.69	23.7 ± 0.7

a The model set is described by the rounded mole percentage of chromophore MAB1 covalently bonded at the PMMA polymer host. All values are average values of five independent models and their corresponding standard deviations. The permittivities ε_λ_, refractive indices in *z*-direction *n*_*z*_ and *r*_33_ values are estimated for a wavelength of λ = 850 nm. The errors in density Δρ, number density Δ*N*_c_, Δε_λ_ and Δ*n*_*z*_ are all less than 0.007 and are therefore omitted.

b The density of pure PMMA (PP) is adapted from our previous study.^17^ The *n*_*z*_ value of the PP can be found in refs 66 and 67 and for PP ε_λ_ = *n*_*z*_^2^ is used.

All *r*_33_ values are plotted in Figure 7 and the calculated optical properties, i.e., the adapted number densities *N*_c_, the permittivities ε_λ_, refractive indices *n*_*z*_, and *r*_33_ values, for the alternative chromophore candidates are provided in the Supporting Information (section S3). *r*_33_ values of C3 in PMMA are adapted from our previous study^17^ as a reference. Figure 7a presents *r*_33_ values at a certain wavelength λ, close to the first absorption transition λ_0_ of the corresponding chromophore. However, no notable absorption is expected at this wavelength (λ > λ_0_ + 300 nm). These *r*_33_ values are an upper limit of the expected EO activity. Figure 7b shows *r*_33_ values for the static limit (ν → 0 or λ­(EO) → ∞) and demonstrate the lower limit of the expected EO activity. The dispersion curves of (hyper)­polarizability values for the different chromophores are included in the Supporting Information (section S3).

**Figure 7:**
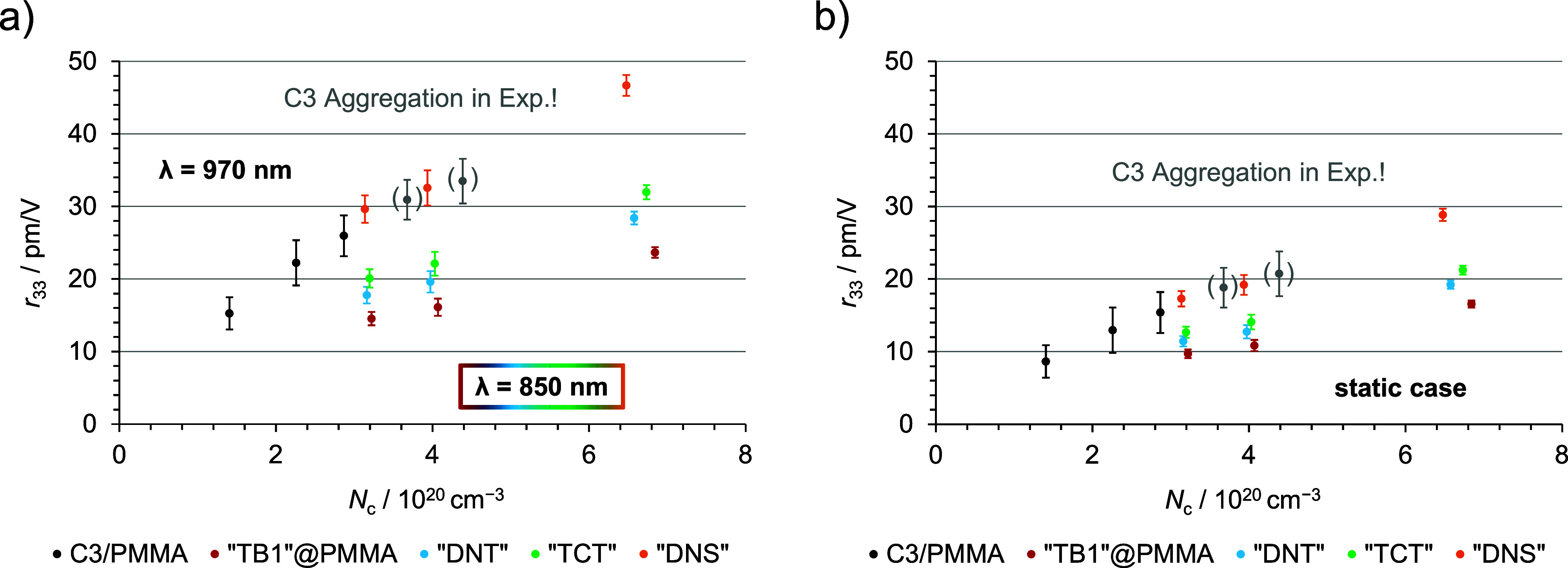
Estimated electro-optic (EO) response under ideal conditions (no effects upon aggregation and simple additive behavior of molecular polarizabilities). For (a) a specified wavelength (possible upper EO activity limit) and for (b) the static limit (ν → 0 or λ­(EO) → ∞) (possible lowest EO activity limit). *r*_33_ values of C3 in PMMA are adapted from our previous study, ref 17 as reference, Ⓒ 2025 The Authors. The gray data points for *N*_c_(C3) > 3 × 10^20^ cm^–3^ represent hypothetical values, as aggregation occurs at these concentrations in experimental settings.

Despite the potential increased number density of chromophores for the covalent incorporation, the expected EO activity for TB1@PMMA is estimated to be approximately half of the EO activity of C3 in PMMA at equivalent concentrations, essentially because of the lower hyperpolarizability of the B1 chromophore (−56% at λ­(EO)). The lower poling efficiency has only a minor negative effect on the EO activity (−10% to −15%). Finally, making small modifications to the TCF acceptor results in more promising EO activities. At this point, the question remains whether the novel DNS chromophore is synthetically available in a laboratory setting. Of all the chromophores that have been investigated, this is the only one that is expected to outperform the previously investigated C3. Nevertheless, besides to the poor performance with regard to molecular hyperpolarizability, covalently bonded chromophore systems are promising due to their augmented long-term stability and broader accessible concentration range for chromophore incorporation. This contrasts with noncovalently incorporated chromophores, such as C3 in PMMA, which undergo aggregation at elevated concentrations^17^ (gray data points for *N*_c_ > 3 × 10^20^ cm^–3^, Figure 7).

## Conclusion and Outlook

This study presents a comprehensive simulation protocol for covalently bonded chromophores. Our previously established methods have been applied, i.e., the glass transition analysis, and the analysis of the poling and relaxation behavior in terms of the molecular order parameter ⟨cos^3^θ⟩.^17^ Elevated glass transition values have been observed for covalently bonded chromophore systems. The simulated glass transition values have been validated through experimental measurements.

Different relaxation conditions have been tested for the simulation models to investigate the long-term alignment stability in detail. Through simulations, we can confirm that long-term stability is enhanced by covalently bonded chromophores. Finally, the ideal electro-optic (EO) activity response, *r*_33_, has been determined. In this context, “ideal” is assumed to be the absence of interaction between the chromophores, resulting in a hyperpolarizability that is the sum of the individual molecular hyperpolarizabilities. This simplified estimation procedure provides a straightforward method for gaining insight into the range of EO activity. However, it is important to note that this additive behavior may not always be accurate, as EO responses can be significantly altered (e.g., amplified, saturated or canceled out) upon interaction with a nonuniform environment as demonstrated in refs 68–71 .

Still, our efficient protocol allowed us to propose novel chromophore candidates, that exhibit an improved EO response. Similarly, novel chromophores and host–guest compositions can be explored using machine learning and/or artificial intelligence to reduce costs and boost innovation in the laboratory. This approach eliminates the need to extensively develop chromophore synthesis pathways in the initial phase.

The presented simulation protocol may be further applied to other popular polymer materials, e.g., poly­(carbonate), poly­(styrene), poly­(urethane), poly­(imide), and poly­(quinoline).^3,21^ The investigation of other polymers and their interactions with the chromophore, the effect on the *T*_g_, the poling efficiency, the alignment stability, and the EO response are subjects that could be addressed by future molecular dynamics studies.

If necessary, an aggregation analysis could be carried out for the high concentration models. Subsequent DFT calculations of the most common aggregates/spatial positioning/orientation may reveal optical properties and EO responses that are more comparable to real-world measurements. However, these extended calculations would also require a significant amount of additional computational and methodological effort. Electro-optical measurements using the Teng–Man setup are planned for the near future.

## Supplementary Material



## Notes

S1 Individual Order Parameter Diagrams with Electric Field Poling under Different Conditions; S2 Illustration of the Phase Behavior; S3 Optical Properties and Electro-Optic Activity of the Studied Chromophore Candidates; S4 ESP Charge Profiles and Steric Profiles of the Studied Chromophore Candidates; S5 ESP Charge Profiles and Steric Profiles of TB1 in Alternative PCM Environments (PDF)

## ⊥

Passed away on January 13, 2023.
