# Supplementary material for: Molecular Dynamics Simulations and Electric Field Poling of Covalently Bonded Chromophores at Poly(methyl Methacrylate)
Source: J Phys Chem B. 2026 Jun 15;130(25):6480–93. doi: 10.1021/acs.jpcb.6c01187 (PMC13312446; doi:10.1021/acs.jpcb.6c01187)
Supplement: Supplementary file 1 [file jp6c01187_si_001.pdf]

Supporting Information: Molecular Dynamics Simulations and Electric Field Poling of Covalently Bonded Chromophores at Poly(methyl methacrylate)

Nils M. Denda,[†,‡] Oguzhan Albayrak,[†,¶] Henning Menzel,[†,¶] Carolin König,[†,§] Peter Behrens,[†,‡,‖] and Andreas M. Schneider[∗,†,‡]

†*Cluster of Excellence PhoenixD (Photonics, Optics, and Engineering – Innovation Across Disciplines), 30167 Hannover, Germany*

‡*Institute of Inorganic Chemistry, Leibniz University Hannover, 30167 Hannover, Germany*

¶*Institute of Technical Chemistry, Technische Universität Braunschweig, 38106 Braunschweig, Germany*

§*Institute of Physical Chemistry and Electrochemistry, Leibniz University Hannover, 30167 Hannover, Germany*

‖*Passed away on January 13, 2023*

E-mail: andreas.schneider@acb.uni-hannover.de

Phone: +49 (0) 511 762 3259. Fax: +49 (0) 511 762 3006

S1 Individual Order Parameter Diagrams with Electric Field Poling under Different Conditions

Figure S1 shows individual order parameter diagrams for poling under different conditions. The individual order parameter curves for the standard poling and relaxation

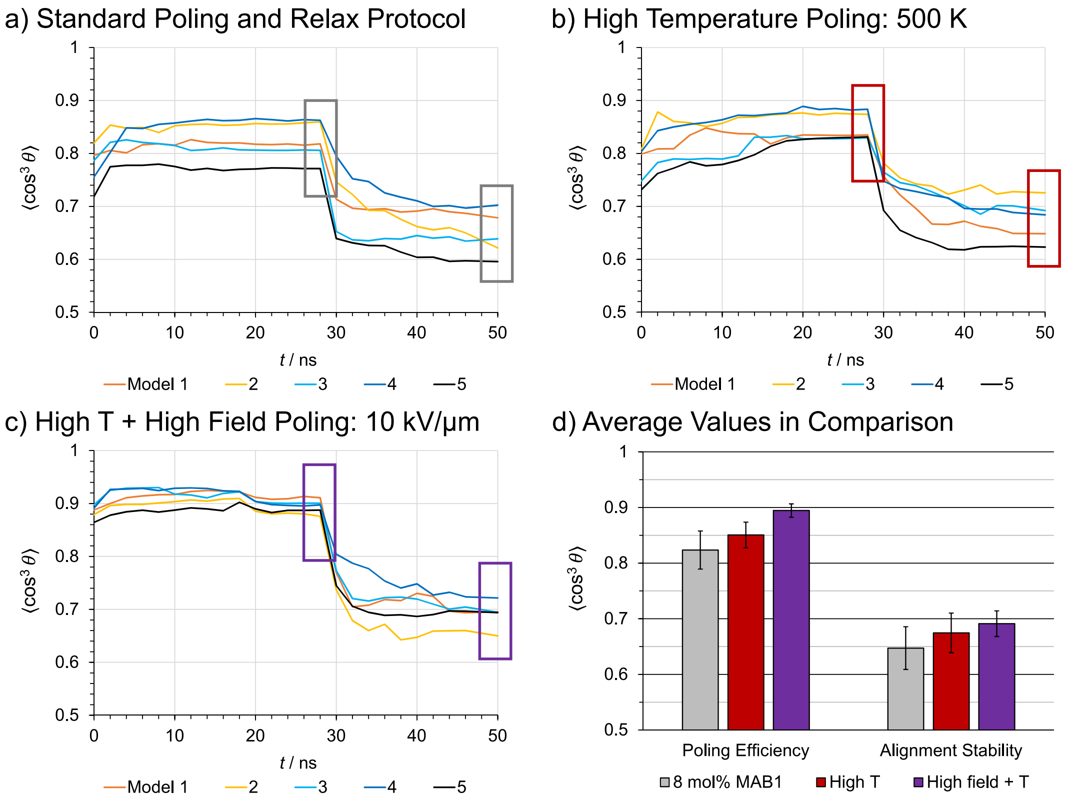

Figure S1: (a-c) Order parameter evolution in the course of different poling conditions from 0 ns to 20 ns for five independent 8 mol% MAB1@PMMA models. (d) Average poling efficiency and alignment stability values (a-c) in direct comparison. The error bars are the respective standard deviations. (a) Standard poling and relaxation protocol: $E = 5\,\mathrm{kV\,\mu m^{-1}}$; $T = 450\,\mathrm{K}$; (b) $E = 5\,\mathrm{kV\,\mu m^{-1}}$; $T = 500\,\mathrm{K}$; (c) $E = 10\,\mathrm{kV\,\mu m^{-1}}$; $T = 500\,\mathrm{K}$. The second poling step (20 ns to 30 ns: $E = 5\,\mathrm{kV\,\mu m^{-1}}$; $T = 300\,\mathrm{K}$) and the relaxation conditions (30 ns to 50 ns: $E = 0\,\mathrm{kV\,\mu m^{-1}}$; $T = 350\,\mathrm{K}$) remain unchanged from the standard poling and relaxation protocol.

simulation protocol (Figure S1a) show a constant level of the order parameter after 10 ns, so that optimal alignment is reached after 10 ns. An elevated poling temperature of 500 K (Figure S1b) facilitates alignment in the electric field and the poling efficiency is slightly en-

hanced (from 75 % to 85 % (std. cond.) to 80 % to 90 % (high T)). The poling efficiency can be increased further under elevated temperatures and a stronger electric field (Figure S1c, $10\,\mathrm{kV\,\mu m^{-1}}$, twice the standard poling field).

However, the order parameter relaxation curves exhibit a comparable behavior to that observed under standard poling and relaxation conditions after all different poling conditions (Figure S1d). The resulting differences are insignificant. The strong electric field of $10\,\mathrm{kV\,\mu m^{-1}}$ induces strong forces on the entire polymer structure. But during the relaxation step, the polymer releases this additional stress, and the chromophores realign elastically. As a result, the order parameter difference from poling efficiency to alignment stability is slightly larger than when poled with lower electric field strengths.

In conclusion, stronger electric fields and adjustments to the simulation protocol, e.g., higher temperatures, should be tested in simulations, especially when the apparent glass transition is elevated. However, since the present study did not observe any significant differences, the previously developed poling and relaxation protocol is applicable.

S2 Illustration of the Phase Behavior

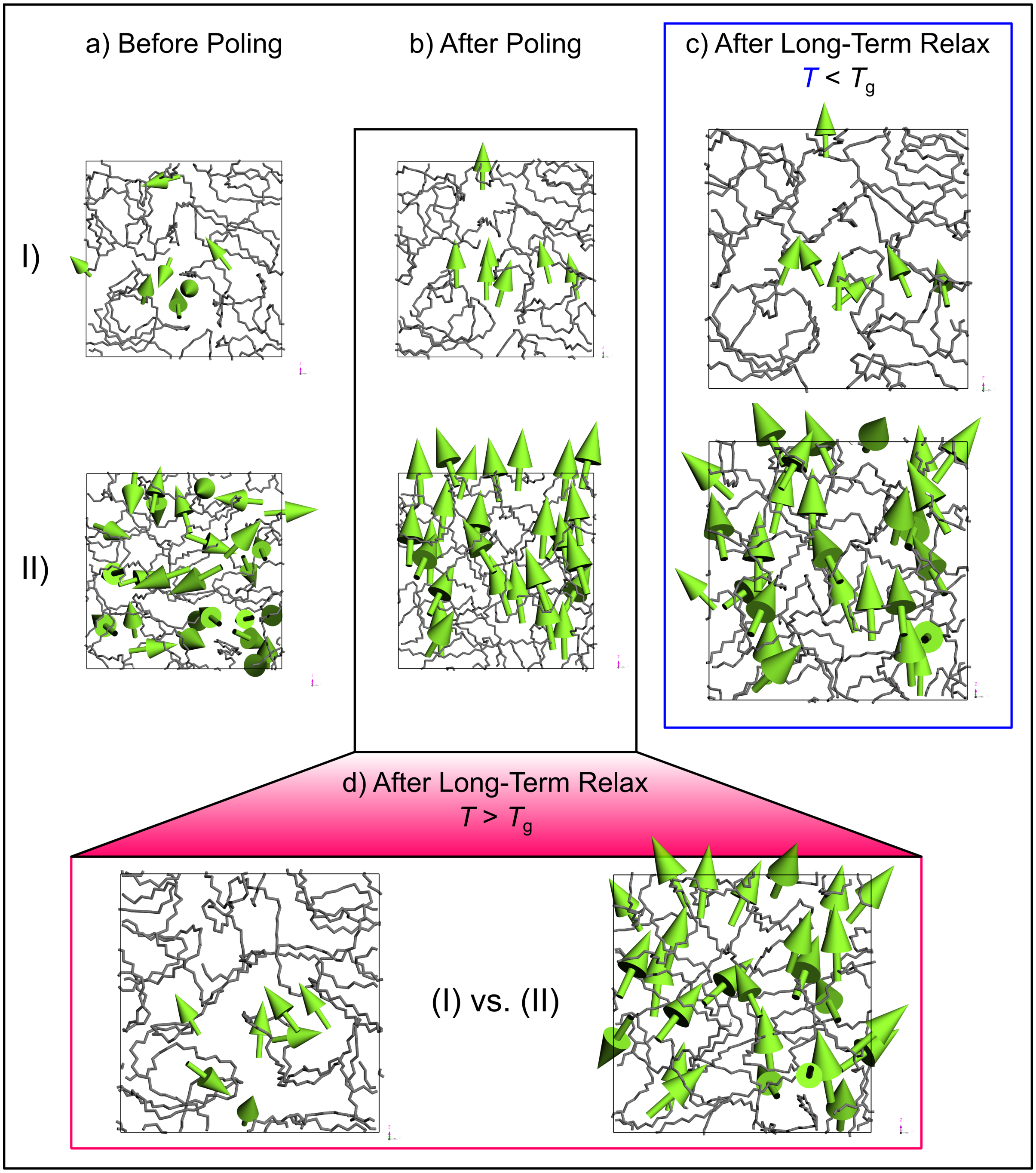

Figure S2: Snapshots of the molecular dynamics simulations of the 2 mol% C3 non-covalent host–guest system (I) and the 8 mol% MAB1 covalently bonded host–guest systems (II) in comparison. In (c) the temperature is $T = 350\,\mathrm{K} < T_g$ and in (d) the temperature is $T = 450\,\mathrm{K} > T_g$.

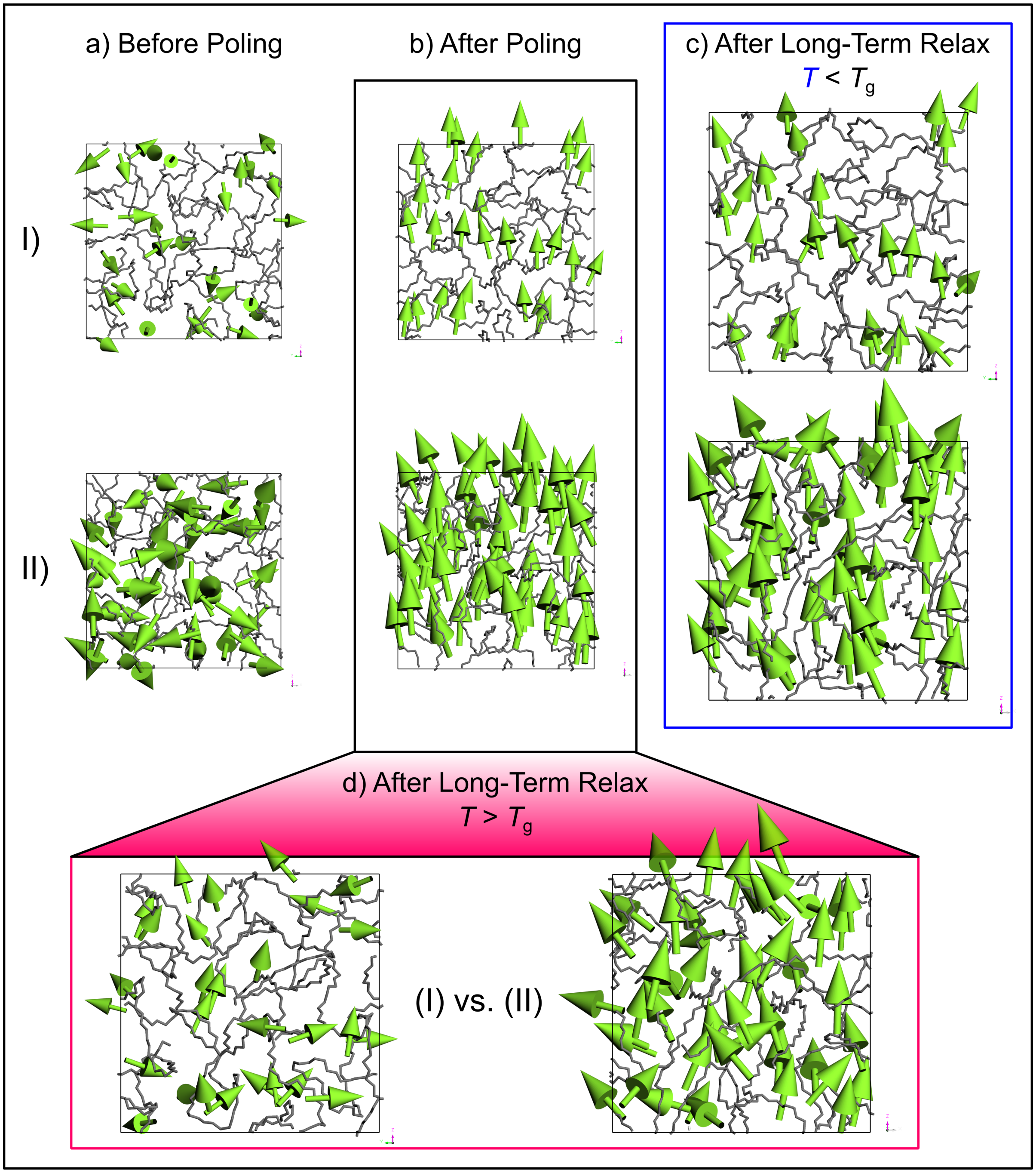

Figure S3: Snapshots of the molecular dynamics simulations of the 9 mol% C3 non-covalent host–guest system (I) and the 17 mol% MAB1 covalently bonded host–guest systems (II) in comparison. In (c) the temperature is $T = 350\,\mathrm{K} < T_\mathrm{g}$ and in (d) the temperature is $T = 450\,\mathrm{K} > T_\mathrm{g}$.

S3 Optical Properties and Electro-Optic Activity of the Studied Chromophore Candidates

In Table S1, an overview of the optical properties of the different studied chromophores is presented. The values of chromophore C3 are adapted as a reference from our previous study[S1] and are provided as a reference. Similarly, the (hyper)polarizabilities of the novel chromophores with alternative acceptor groups (DNT, TCT, DNS) are presented as a reference. The most important value for the assessment of electro-optical performance is the hyperpolarizability along the molecular dipole moment $\beta_{||}$ (usually the molecule is aligned with its dipole moment into the z-direction of the molecular coordinate system). In the following, the values for MAB1, B1, and TB1 are discussed in detail. The other acceptor group modifications of DNT, TCT, and DNS are discussed in the main manuscript.

The polarizabilities and hyperpolarizabilities are presented for two cases: the values for the minimum wavelength (or maximum frequency) of the electro-optic (EO) experiment ($\lambda(\mathrm{EO}) > \lambda_0 + 300\,\mathrm{nm}$), before notable absorption at λ_0 begins; and the static case (with $\nu = 0$ or $\lambda \to \infty$), respectively. The former case may represent an upper limit of expected EO activity under ideal conditions, while the latter case corresponds to the lower limit of expected EO activity.

DFT calculations for three variants of the B1 chromophore were carried out: First, the complete chromophore with the methyl methacrylate (MMA) unit (fully saturated), i.e., MAB1 was investigated (cf. to Figure 1c, main manuscript, but the C=C double bond of MMA is fully saturated with hydrogen). Secondly, the isolated B1 chromophore before condensation with the MMA unit, i.e., B1 was examined (cf. to Figure 1b, main manuscript). Finally, the truncated B1 (TB1), in which the methoxy group of the chromophore side chain is assigned to the poly(MMA) backbone, i.e., TB1 was calculated (cf. to Figure 1d, main manuscript). These three variants of the B1 chromophore were examined to investigate, the impact of the polymer backbone (MMA unit) and the hydroxy propoxide side chain of the

Table S1: Chromophore Properties, Polarizabilities[a] α and Hyperpolarizabilities[b] β Dependent on the Wavelength of the Electro-Optic Experiment[c] λ(EO)

Chromophore	C3[d]	MAB1[e]	B1	TB1	DNT	TCT	DNS
Formula	$C_{27}H_{29}N_5O_2$	$C_{29}H_{34}N_4O_4$	$C_{25}H_{28}N_4O_3$	$C_{24}H_{26}N_4O_2$	$C_{22}H_{26}N_4O_6$	$C_{24}H_{26}N_4OS$	$C_{22}H_{26}N_4O_5S$
M / g mol^{-1}	455.6	502.6	432.5	402.5	442.5	418.6	458.5
λ_0 / nm	606	485	471	472	486	490	506
λ(EO) / nm	970	850	850	850	850	850	850
α_{xx}	43.4	65.2	42.5	43.7	43.9	57.0	46.9
α_{yy}	66.3	55.9	68.2	64.2	64.6	62.8	79.2
α_{zz}	240.2	175.3	158.1	154.3	166.5	164.4	173.2
$\beta_{\|\|}$	641.2	277.4	272.3	283.8	356.4	403.1	607.2
β_{tot}	1068.6	462.4	453.8	473.0	594.0	671.8	1012.0
Values for the Static Limit Below ($\nu \to 0$ or $\lambda(EO) \to \infty$)							
α_{xx}	45.3	68.3	45.3	45.6	45.7	58.2	48.6
α_{yy}	69.9	58.0	69.5	65.3	65.8	63.5	77.5
α_{zz}	195.0	152.2	137.2	133.4	142.0	140.6	145.3
$\beta_{\|\|}$	345.0	173.2	183.6	179.9	215.9	239.8	330.6
β_{tot}	574.9	288.7	306.1	299.8	359.9	399.7	551.1

[a] in 10^{-24} esu; [b] in 10^{-30} esu; [c] The wavelength of the electro-optic experiment λ(EO) is determined based on the wavelength λ_0, i.e., the lowest absorption energy of the corresponding chromophore ($\lambda(EO) > \lambda_0 + 300$ nm); [d] Properties of C3 are adapted from ref S1 as reference, © 2025 The Authors; [e] the methyl methacrylate unit is fully saturated for the DFT calculation.

chromophore on the optical properties and their contribution to the (hyper)polarizabilities.

The wavelength of the first absorption λ_0 of MAB1 compared to (T)B1 shows a slight blue shift ($\Delta\lambda \approx 10\,\mathrm{nm}$). MAB1 shows a slightly larger polarizability in x- and z-direction compared to the isolated chromophores B1 or TB1 ($\Delta\alpha_{xx}$ and $\Delta\alpha_{zz} \approx +20 \times 10^{-24}$ esu or $\Delta\alpha_{xx} = +50\,\%$ and $\Delta\alpha_{zz} = +13\,\%$). The MMA group at the MAB1 with the propyloxy side chain is perpendicular to the xz-plane of the molecular coordinate system (z-axis = dipole moment), so that the carbonyl group of the MMA can contribute to the total polarizability in z-direction. Out of the xz-plane (along the y-axis), the polarizability is lowered compared to the isolated chromophores (T)B1 ($\Delta\alpha_{yy} \approx -10 \times 10^{-24}$ esu or $\Delta\alpha_{yy} = -18\,\%$), because of the low polarizable propyloxy chain in x- or z-direction, which distorts the electron density distribution away from the main charge transfer axis (z-axis). The oxygen atoms of the side chain are slightly contributing to the polarizabilities in the x-direction. The energy minimized structure of MAB1 is displayed in Figure S4.

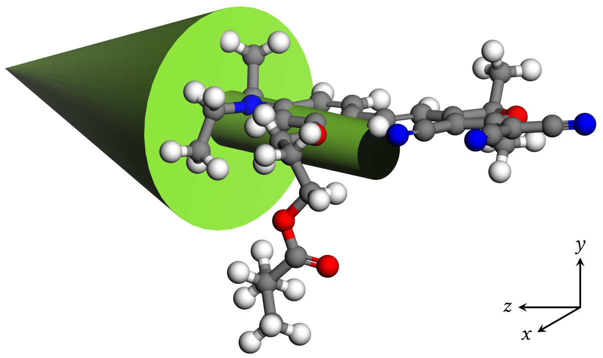

Figure S4: The energy-minimized structure of MAB1 was calculated with DFT methods.

These polarizability values show, that the side group/polymerizable unit of the chromophore may be treated separately. In the z-direction, there is no significant contribution to the polarizability (+13 %), whereas in the x-direction the polarizability of the MMA unit is visible in the case of MAB1 (+50 %). In order to facilitate DFT calculations and to make the calculation of the r_{33} value more straightforward, the chromophore is separated from the polymerizable unit (MAB1 $\rightarrow$ B1 $\rightarrow$ TB1). This is done for two reasons. First, the

polarizability of the polymer repeat unit is already implicitly included in the calculation procedure because of the utilization of polarizable continuum model calculations in the DFT calculations of the chromophores, since the chromophores are “dissolved” in the polymer (for details see ref S1). Second, the treatment of less atoms in DFT calculations is beneficial, especially if large polymerizable units or larger oligomers of covalently bonded chromophores to the polymer chain should be treated.

For the straightforward calculation of the EO effect, only the total hyperpolarizability β_{tot}, arising from the tensor element parallel to the dipole moment $\beta_{||}$ is important. For the present case $\beta_{\mathrm{tot}} = \frac{5}{3} \times \beta_{||}$ holds true, because the charge transfer is unidirectional and parallel to the molecular dipole moment.[S2] Therefore, the discussion of the first hyperpolarizability is limited to the tensor element parallel to the dipole moment $\beta_{||}$.

The hyperpolarizabilities of MAB1, B1 and TB1 are not significantly different ($\Delta\beta_{||} <$ 6 %). The side chain/polymerizable unit has a marginally negative effect on $\beta_{||}$. At 850 nm $<$ 4 % for B1 and MAB1 compared to TB1. And for the static case $<$ 6 % for MAB1 compared to B1 or TB1, because the electron density of the polymerizable unit is located outside the plane of the main charge transfer and does not contribute to the electron transfer along the dipole moment (compare Figure S4). This result highlights, that the side chain with the MMA unit may contribute to the polarizabilities α, but the hyperpolarizability $\beta_{||}$ is more sensitive to the electron transfer along the dipole moment and not to changes in the movement of electron density at the side chain.

The neglect of the $-OCH_2-$ group (or attribution to the polymer backbone, see Figure 1c, main manuscript, i.e., the transition from B1 to TB1) has also only a small impact on the hyperpolarizabilities (compare $\beta_{||}$ values of B1 with TB1, $\Delta\beta_{||} < 4$ % or < 2 % for the 850 nm or the static case, respectively).

The presented β values are the result of a DFT energy minimization. Consequently, the very small effect on $\beta_{||}$ is dependent on the local arrangement of the side group/polymerizable unit. Again, we are aiming at a straightforward simulation and calculation

Table S2: Polarizabilities[a] α and Hyperpolarizabilities[b] β Calculated with Different Range-Separated DFT Functionals[c]

Method	$\lambda = 850\,\text{nm}$		static	
	α_{zz}	$\beta_{\|\|}$	α_{zz}	$\beta_{\|\|}$
LC-BLYP[S4,S5]	141.5	305.4	125.3	202.6
M11[S6]	148.1	298.6	129.5	191.1
ωB97X[S7]	146.0	294.2	128.5	191.8
CAM-B3LYP[S8]	154.3	283.8	133.4	179.9
Avg.	147.5	295.5	129.2	191.4
Abs. Std. Dev.	4.6	7.8	2.9	8.0
Rel. Std. Dev. / %	3.1	2.6	2.2	4.2
Min-Max / %	8.7	7.3	6.3	11.9

[a]in 10^{-24} esu; [b]in 10^{-30} esu; [c]Molecular structures were first minimized in energy using the B3LYP[S9,S10]/aug-cc-pVTZ[S11–S13] method. Next, the CPHF calculations[S14–S16] were performed to obtain the different (hyper)polarizabilities. All calculations were performed in a polarizable continuum model.[S17–S19] See the main manuscript for details.

procedure for the assessment of the EO activity. Therefore, it is not beneficial to optimize large oligomers on DFT level to obtain β values, that may be as accurate as possible to low energetic states, because the actual conformation of the covalently bonded chromophores depend on the local structure in the host polymer, which is dominated by the polymer chain interactions and/or the electric field. Thus, we propose to cut off the chromophore from the polymer/polymerizable unit, in order to treat the EO active part with high-level DFT methods. This procedure needs to be revisited when employing other chromophores with large or highly polarizable side-groups/polymerizable units (e.g., benzene, polar functional groups, etc.), which may align in the electric field and may significantly contribute to the total (hyper)polarization of the material or are in resonance with the main charge transfer process.

Table S2 shows average values and standard deviations of calculated polarizabilities and hyperpolarizabilities of TB1, which were calculated using different recommended DFT functionals.[S3] In the original article by Johnson *et al.*[S3], there have been also experimental

investigations of hyperpolarizabilities employing Hyper-Rayleigh scattering, showed that the DFT calculated results for static hyperpolarizabilities deviated by up to 25 %, revealing a spread of values of approximately 20 %. Although, these deviations seem to be large, theoretical estimations are indispensable, because especially the hyperpolarizability for the Pockels effect $\beta(-\omega, 0, \omega)$ is not available experimentally[S3] and theoretical studies support experimental investigators in developing novel, promising chromophore candidates. Paschoal and Dos Santos[S20] found the following errors and uncertainties: Errors for DFT calculated static hyperpolarizabilities of up to 30 %, for dynamic hyperpolarizabilities an error of up to 35 %, and experimental uncertainties of up to 20 %.

In the present study, the examined chromophores are much smaller than those examined by Johnson *et al.*[S3] and we cannot provide experimental measurements. However, if the reliability of the DFT calculated results is assumed, as demonstrated by Johnson *et al.*[S3], then an average uncertainty of approximately 5 % can be attributed to the calculated hyperpolarizabilities. This estimated uncertainty for DFT calculated hyperpolarizabilities demonstrates that truncating the side group/polymerizable unit does not introduce a significant error in the present case.

Figure S5 shows the dispersion of the (hyper)polarizabilities of the different chromophore candidates.

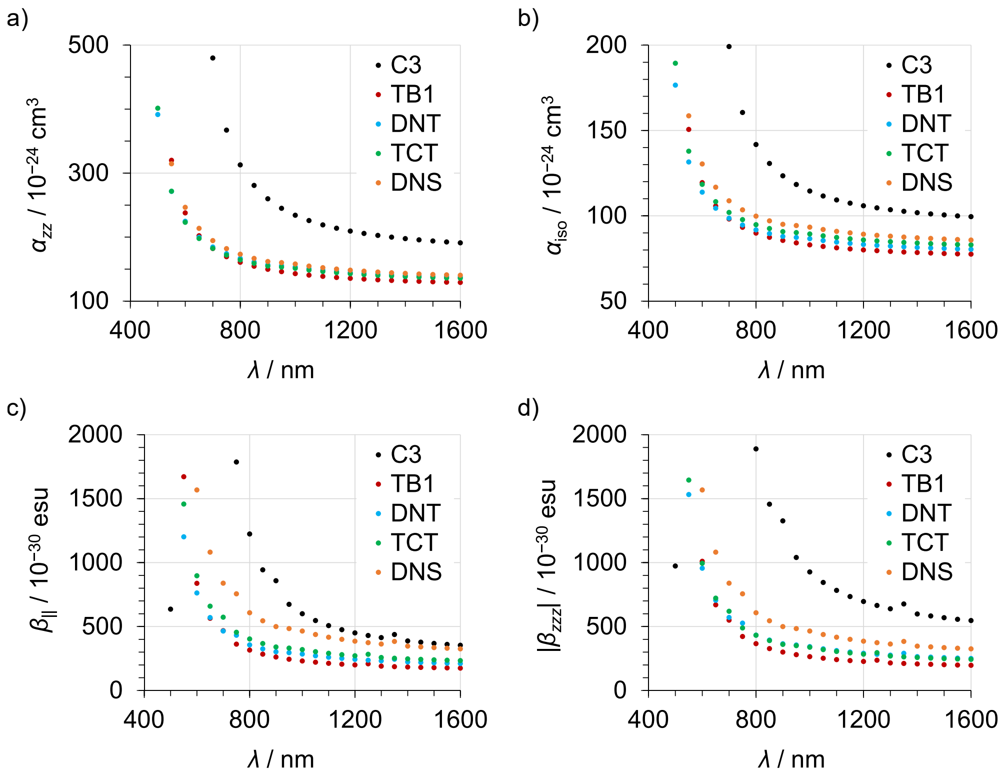

Figure S5: Polarizabilities (a,b) and hyperpolarizabilities (c,d) of all chromophores studied in this paper.

Table S3 summarizes the EO activity values of the alternative chromophore candidates with modified acceptor groups.

Table S3: Overview of Alternative Chromophore Candidates and the Electro-Optic Activity (Values After Relaxation)[a]

Model Set	N_c / $10^{20}\,cm^{-3}$	ε_λ	n_z	r_{33} / $pm\,V^{-1}$
PP[b]	–	2.19	1.48	–
Values for $\lambda(EO) = 850\,nm$				
6 mol% "TB1"	3.22	2.30	1.57	14.5 ± 0.9
8 |	4.07	2.34	1.60	16.1 ± 1.2
17 |	6.84	2.46	1.69	23.7 ± 0.7
6 mol% "DNT"	3.16	2.29	1.58	17.8 ± 1.1
8 |	3.97	2.33	1.60	19.6 ± 1.5
17 |	6.58	2.44	1.69	28.4 ± 0.9
6 mol% "TCT"	3.20	2.32	1.58	20.1 ± 1.3
8 |	4.03	2.37	1.61	22.1 ± 1.6
17 |	6.73	2.50	1.70	32.0 ± 1.0
6 mol% "DNS"	3.14	2.31	1.58	29.6 ± 1.9
8 |	3.94	2.36	1.61	32.6 ± 2.4
17 |	6.48	2.48	1.70	46.7 ± 1.4
Values for the Static Limit Below ($\nu \to 0$ or $\lambda(EO) \to \infty$)				
6 mol% "TB1"	3.22	2.27	1.55	9.7 ± 0.6
8 |	4.07	2.31	1.57	10.8 ± 0.8
17 |	6.84	2.41	1.65	16.5 ± 0.5
6 mol% "DNT"	3.16	2.26	1.55	11.4 ± 0.7
8 |	3.97	2.30	1.57	12.7 ± 0.9
17 |	6.58	2.38	1.64	19.2 ± 0.6
6 mol% "TCT"	3.20	2.29	1.56	12.7 ± 0.8
8 |	4.03	2.33	1.58	14.1 ± 1.0
17 |	6.73	2.44	1.66	21.2 ± 0.6
6 mol% "DNS"	3.14	2.28	1.55	17.3 ± 1.1
8 |	3.94	2.32	1.57	19.2 ± 1.4
17 |	6.48	2.41	1.65	28.8 ± 0.8

[a]The model set is described by the rounded mole percentage of chromophore MAB1 covalently bonded at the PMMA polymer host. All values are average values of five independent models and their corresponding standard deviations. The model set density ρ and order parameter $\langle \cos^2\theta \rangle$ and $\langle \cos^3\theta \rangle$ are adapted from the MAB1 simulations (see main manuscript, not tabulated here). The errors in number density ΔN_c, permittivity $\Delta\varepsilon_\lambda$, and refractive index in z-direction Δn_z are all less than 0.007 and are therefore omitted; [b]The n_z value of the pure PMMA (PP) can be found in refs S21,S22.

S4 ESP Charge Profiles and Steric Profiles of the Studied Chromophore Candidates

All atomic partial charges were obtained by electrostatic potential (ESP) fitting as described in the main manuscript.[S23,S24] Figure S6 displays the atom labels and the modifications of the different chromophore candidates. Figure S7 shows the ESP charge profile of

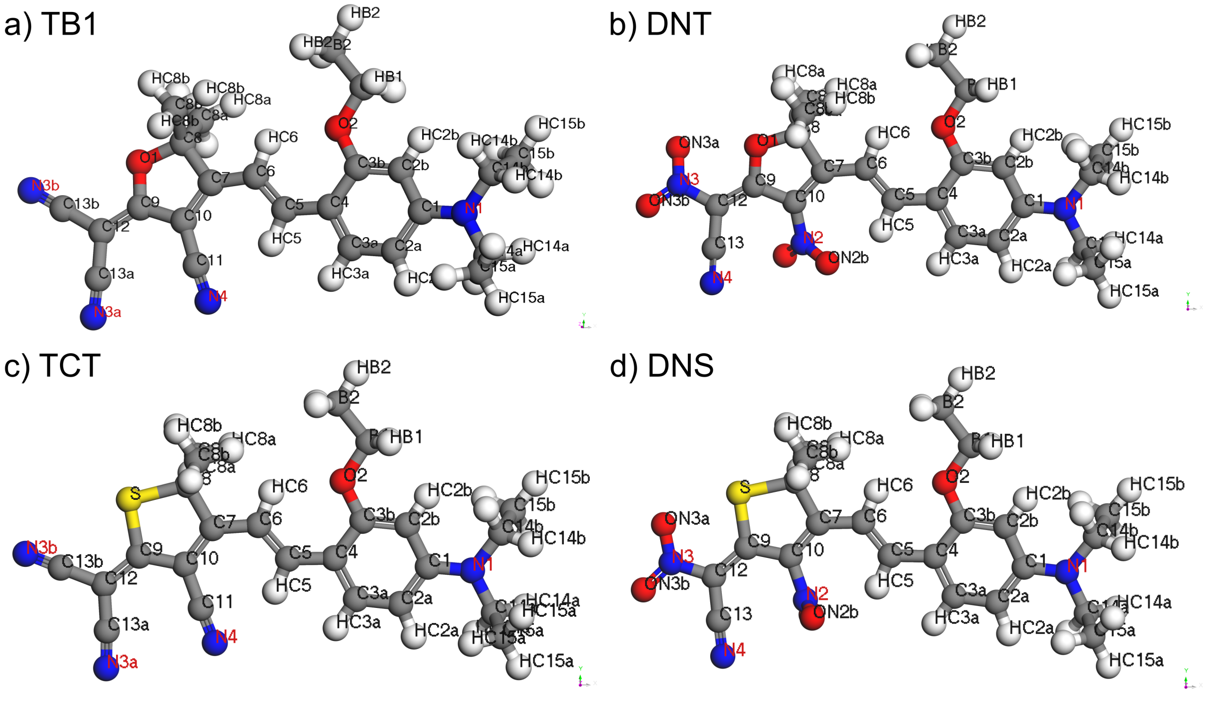

Figure S6: Atom labels of the studied chromophores. Modifications of TB1 have been performed at position O1 (O1 → S) or at positions C10 and C12 (−CN → $-NO_2$). Element coloring: N blue, O red, S yellow, C gray, H white.

the different chromophores. The color of each atom indicates its atomic charge. The acceptor group is located on the left side (including C7), and a reference presentation in element coloring is provided in Figure S6. The charge profile shows that charges slightly decrease in the five-membered ring of the acceptor group, from TB1 to DNT to TCT to DNS. The sulfur-containing acceptor groups (TCT and DNS) have lower charges in the five-membered ring than TB1 and DNT. Furthermore, it is evident that the remainder of the chromophore system is remains largely unaffected by the modifications in the acceptor

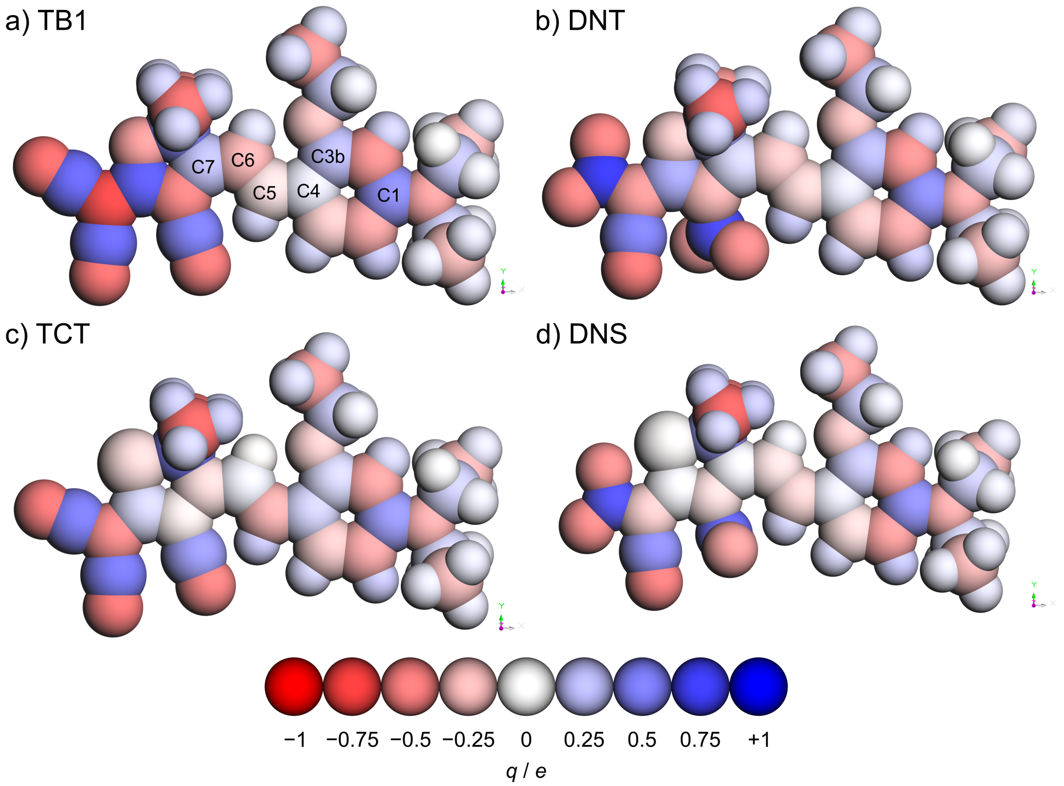

Figure S7: ESP charge profile of the studied chromophores. Results of an energy minimization with B3LYP/aug-cc-pVTZ, PCM = diethylamine (DEA).

group. The π electron bridge (C6-C1) is marginally impacted (on atoms C6-C4), while the side chain (at C3b) and the donor group (atoms beyond C1) appears to be unaltered. Most importantly, the charge distribution at the outer acceptor group atoms remains almost the same, suggesting that intermolecular interactions should be comparable. The explicit charges for direct comparison are listed below, and a more in-depth comparison will be provided later.

Figures S8-S10 show the steric profiles of the different acceptor group modifications, as well as the variation in dipole moment. The dipole moments obtained by an energy minimization with B3LYP/aug-cc-pVTZ (PCM: diethylamine) are 27.7 D, 30.2 D, 26.9 D and 27.4 D for TB1, DNT, TCT, and DNS, respectively. The introduction of nitro groups increases the dipole moment whereas the introduction of the sulfur decreases the dipole moment. The dipole moment difference of 2.5 D (+9 %) by comparing DNT with TB1

a) Superposition of TB1 (gray + element coloring: C gray, N blue, O red, H white) and DNT (magenta)

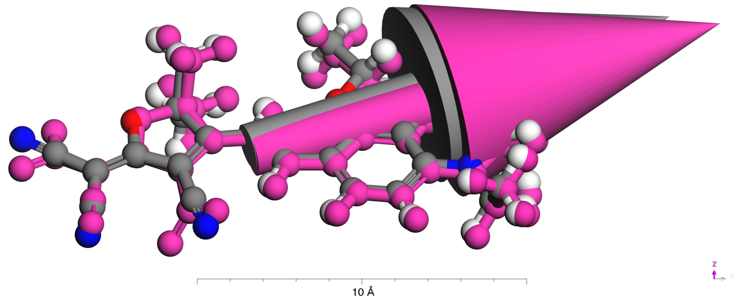

b) Superposition of TB1 and DNT (side view)

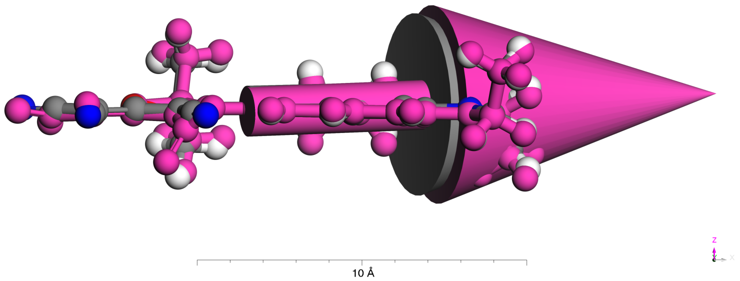

Figure S8: Dipole moments and steric profiles of TB1 and DNT. Results of an energy minimization with B3LYP/aug-cc-pVTZ, PCM = diethylamine (DEA).

a) Superposition of TB1 and TCT (magenta)

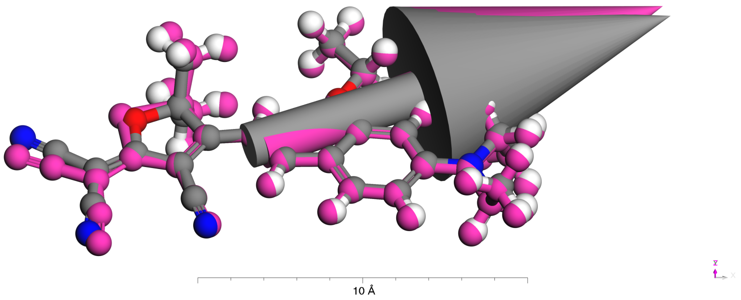

b) Superposition of TB1 and TCT (side view)

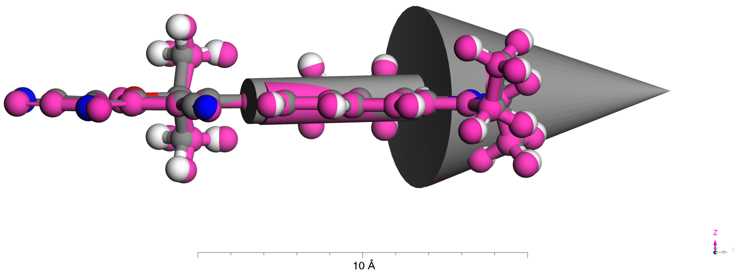

Figure S9: Dipole moments and steric profiles of TB1 and TCT. Results of an energy minimization with B3LYP/aug-cc-pVTZ, PCM = diethylamine (DEA).

a) Superposition of TB1 and DNS (magenta)

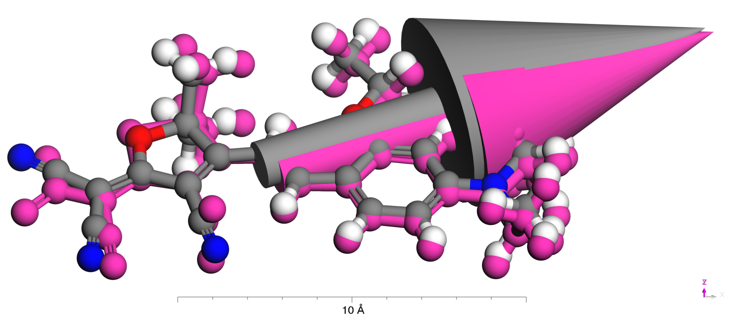

b) Superposition of TB1 and DNS (side view)

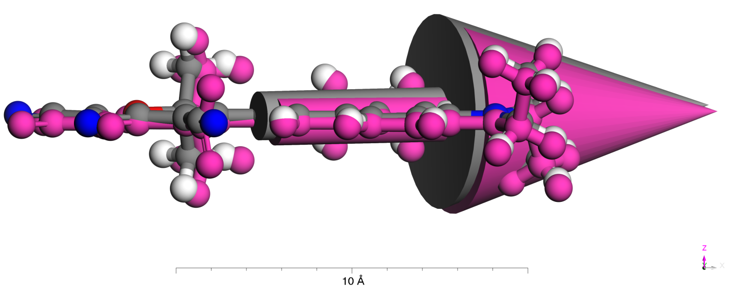

Figure S10: Dipole moments and steric profiles of TB1 and DNS. Results of an energy minimization with B3LYP/aug-cc-pVTZ, PCM = diethylamine (DEA).

appears large, but considering the size of the entire chromophore system, the effects arising from the dipole moment differences are assumed to be small for the MD simulations. The dipole moment differences between TB1 and the sulfur containing chromophores are smaller than 3 % and are assumed to be negligible.

The ball and stick models of the different chromophores show that the nitro groups and the sulfur in the five-membered ring need more space than the cyano groups and the oxygen atom in the five-membered ring. However, this additional required space deviates from the original sterical demand of the TB1 chromophore only to a small extent. The nitro group at the vinyl end of the acceptor group of DNT and DNS is aligned in the main plane of the molecule. The other nitro group of DNT and DNS at the five-membered ring twists out of the main plane but requires less space than the dimethyl group on the opposite side in proximity to the heteroatom in the five-membered ring. As illustrated in the side view (e.g., Figure S10b)), the nitro groups exhibit a lower degree of sterical demand in

comparison to the dimethyl group.

Table S4 summarizes the van der Waals surface areas and volumes of the chromophores. The largest differences in surface area and volume are between TB1 and DNS $\left(\Delta A = 17.7\,\text{Å}^2 \text{ and } \Delta V = 20.0\,\text{Å}^3\right)$. The smallest and largest atoms in these molecules are hydrogen and sulfur, with van der Waals surface areas and volumes of $A_\text{H} = 18.0\,\text{Å}^2$, $V_\text{H} = 7.2\,\text{Å}^3$ and $A_\text{S} = 40.6\,\text{Å}^2$, $V_\text{S} = 24.0\,\text{Å}^3$, respectively. The change in surface area due to chromophore modification is less than the surface area of one hydrogen atom, and the change in enclosed volume is less than the volume of one sulfur atom (or approximately three hydrogen atoms). The relative surface area and volume changes for TB1 to DNS are $\Delta A_\text{rel.} = 3.8\,\%$ and $\Delta V_\text{rel.} = 5.2\,\%$, respectively. These values quantitatively demonstrate that chromophore modifications have only a minor impact on the overall steric demand, indicating that substantial differences in phase behavior can be excluded.

Table S4: Van der Waals Surface Areas and Volumes of the Studied Chromophores[a]

Chromophore	Surface / Å^2	Volume / Å^3
TB1	462.5	384.7
DNT	473.8	396.1
TCT	466.9	393.7
DNS	480.2	404.7

[a]Grid interval: 0.15 Å, calculated with Materials Studio Visualizer[S25] after energy minimization with B3LYP/aug-cc-pVTZ, PCM = diethylamine (DEA) in Gaussian 16.[S26]

Table S5 contains the partial atomic charges of the different chromophores. Table S6 contains the deviations of the atomic charge parameters between the chromophores. Finally, Table S7 summarizes the differences and deviations of the atomic charge parameters. In order to maintain clarity and simplicity in the discourse, the initial focus will be on Table S7 to present an overview of the atomic charge parameter differences and deviations. The upper half of Table S7 reveals the minimum, maximum, and average differences in the charge parameters of comparable atoms. Table S6 provides a complete presentation

Table S5: ESP Charges of the Studied Chromophores (TB1, TCT, DNS, DNT) in Comparison[a]

Atom	TB1	Atom	TCT	Atom	DNS	Atom	DNT
B1	0.21	B1	0.20	B1	0.20	B1	0.22
B2	−0.42	B2	−0.40	B2	−0.38	B2	−0.40
C1	0.47	C1	0.42	C1	0.45	C1	0.46
C10	−0.50	C10	−0.05	C10	−0.12	C10	−0.29
C11	0.53	C11	0.40	–	–	–	–
C12	−0.75	C12	−0.47	C12	−0.19	C12	−0.40
C13a	0.62	C13a	0.55	C13	0.46	C13	0.51
C13b	0.62	C13b	0.54	–	–	–	–
C14a	0.18	C14a	0.17	C14a	0.15	C14a	0.16
C14b	0.22	C14b	0.19	C14b	0.19	C14b	0.16
C15a	−0.25	C15a	−0.25	C15a	−0.27	C15a	−0.26
C15b	−0.28	C15b	−0.29	C15b	−0.28	C15b	−0.29
C2a	−0.36	C2a	−0.35	C2a	−0.39	C2a	−0.34
C2b	−0.44	C2b	−0.37	C2b	−0.39	C2b	−0.44
C3a	−0.19	C3a	−0.20	C3a	−0.13	C3a	−0.20
C3b	0.25	C3b	0.15	C3b	0.21	C3b	0.26
C4	0.07	C4	0.17	C4	0.06	C4	0.07
C5	−0.11	C5	−0.31	C5	−0.16	C5	−0.15
C6	−0.29	C6	0.05	C6	−0.10	C6	−0.21
C7	0.24	C7	−0.13	C7	0.03	C7	0.14
C8	0.53	C8	0.58	C8	0.44	C8	0.40
C8a	−0.49	C8a	−0.60	C8a	−0.57	C8a	−0.42
C8b	−0.56	C8b	−0.61	C8b	−0.62	C8b	−0.61
C9	0.67	C9	0.15	C9	0.02	C9	0.34
HB1	0.05	HB1	0.05	HB1	0.05	HB1	0.05
HB1	0.05	HB1	0.05	HB1	0.05	HB1	0.05
HB2	0.13	HB2	0.12	HB2	0.11	HB2	0.13
HB2	0.13	HB2	0.12	HB2	0.11	HB2	0.13
HB2	0.12	HB2	0.11	HB2	0.11	HB2	0.11
HC14a	0.05	HC14a	0.05	HC14a	0.06	HC14a	0.06
HC14a	0.01	HC14a	0.01	HC14a	0.02	HC14a	0.02
HC14b	0.05	HC14b	0.06	HC14b	0.06	HC14b	0.07
HC14b	0.00	HC14b	0.01	HC14b	0.01	HC14b	0.02
HC15a	0.07	HC15a	0.07	HC15a	0.07	HC15a	0.07
HC15a	0.07	HC15a	0.07	HC15a	0.08	HC15a	0.07
HC15a	0.08	HC15a	0.08	HC15a	0.08	HC15a	0.08
HC15b	0.08	HC15b	0.09	HC15b	0.08	HC15b	0.09
HC15b	0.08	HC15b	0.08	HC15b	0.08	HC15b	0.09
HC15b	0.07	HC15b	0.08	HC15b	0.08	HC15b	0.08
HC2a	0.20	HC2a	0.21	HC2a	0.21	HC2a	0.20
HC2b	0.16	HC2b	0.14	HC2b	0.14	HC2b	0.16
HC3a	0.16	HC3a	0.16	HC3a	0.14	HC3a	0.16
HC5	0.16	HC5	0.20	HC5	0.16	HC5	0.21
HC6	0.10	HC6	−0.02	HC6	0.03	HC6	0.09
HC8a	0.14	HC8a	0.18	HC8a	0.18	HC8a	0.13
HC8a	0.16	HC8a	0.18	HC8a	0.17	HC8a	0.15
HC8a	0.14	HC8a	0.15	HC8a	0.14	HC8a	0.13
HC8b	0.16	HC8b	0.15	HC8b	0.16	HC8b	0.17
HC8b	0.18	HC8b	0.18	HC8b	0.19	HC8b	0.19
HC8b	0.16	HC8b	0.18	HC8b	0.20	HC8b	0.19
N1	−0.37	N1	−0.33	N1	−0.33	N1	−0.34
N3a	−0.58	N3a	−0.56	N4	−0.52	N4	−0.55
N3b	−0.60	N3b	−0.56	N3	0.72	N3	0.82
N4	−0.54	N4	−0.51	N2	0.63	N2	0.77
O1	−0.39	S	−0.14	S	−0.01	O1	−0.19
O2	−0.23	O2	−0.18	O2	−0.20	O2	−0.24
				ON2a	−0.37	ON2a	−0.42
				ON2b	−0.37	ON2b	−0.46
				ON3a	−0.45	ON3a	−0.51
				ON3b	−0.48	ON3b	−0.50

[a] q in e; Energy minimization with B3LYP/aug-cc-pVTZ, PCM = diethylamine.

Table S6: Deviations in ESP Charges of the Studied Chromophores (TB1, TCT, DNS, DNT)[a]

Atom	Unsigned Absolute Deviation / e			Unsigned Relative Deviation / %		
	TB1−TCT	TB1−DNS	TB1−DNT	TB1−TCT /TB1	TB1−DNS /TB1	TB1−DNT /TB1
B1	0.01	0.01	0.00	4.4	7.0	2.3
B2	0.02	0.05	0.02	4.7	10.9	5.5
C1	0.05	0.02	0.01	11.4	4.9	2.2
C10	0.45	0.38	0.21	89.1	75.8	42.7
C11	0.13	–	–	24.3	–	–
C12	0.28	0.56	0.35	37.1	74.9	46.5
C13a / C13	0.07	0.16	0.10	11.1	25.5	17.0
C13b	0.09	–	–	14.0	–	–
C14a	0.02	0.03	0.03	9.6	16.9	14.5
C14b	0.04	0.03	0.07	16.9	15.6	29.8
C15a	0.01	0.01	0.00	2.3	4.6	0.4
C15b	0.01	0.01	0.02	4.7	2.4	6.1
C2a	0.00	0.04	0.02	0.4	10.7	4.9
C2b	0.07	0.05	0.00	16.6	11.1	0.9
C3a	0.00	0.06	0.01	1.1	31.9	4.0
C3b	0.10	0.05	0.01	41.3	19.1	4.0
C4	0.10	0.00	0.00	153.8	5.8	3.0
C5	0.20	0.05	0.05	184.8	47.1	43.6
C6	0.34	0.19	0.09	116.7	64.4	29.8
C7	0.37	0.21	0.10	156.0	88.9	39.7
C8	0.05	0.08	0.12	9.9	16.0	23.0
C8a	0.11	0.08	0.07	21.9	15.7	14.0
C8b	0.05	0.06	0.05	8.6	10.5	9.0
C9	0.52	0.65	0.33	77.7	97.1	49.4
HB1	0.00	0.00	0.00	5.9	5.7	2.4
HB1	0.00	0.00	0.00	2.4	4.2	6.6
HB2	0.01	0.02	0.01	8.7	15.1	4.7
HB2	0.01	0.02	0.01	8.8	14.3	5.1
HB2	0.00	0.01	0.01	2.0	6.9	5.1
HC14a	0.00	0.01	0.01	3.9	15.8	19.5
HC14a	0.00	0.01	0.00	34.6	78.9	44.3
HC14b	0.01	0.01	0.02	20.8	19.3	48.1
HC14b	0.01	0.01	0.02	440.6	408.7	861.2
HC15a	0.00	0.00	0.00	1.5	7.1	2.2
HC15a	0.00	0.00	0.00	4.4	3.3	0.7
HC15a	0.00	0.01	0.00	0.2	8.9	5.2
HC15b	0.00	0.00	0.01	5.0	2.6	11.7
HC15b	0.01	0.01	0.01	9.0	6.9	13.8
HC15b	0.00	0.00	0.01	6.3	4.6	10.2
HC2a	0.00	0.01	0.00	1.0	5.2	0.0
HC2b	0.02	0.02	0.00	14.0	12.0	0.2
HC3a	0.01	0.02	0.00	3.1	12.3	0.1
HC5	0.04	0.00	0.05	26.1	1.3	29.9
HC6	0.12	0.07	0.02	115.7	66.7	16.6
HC8a	0.04	0.04	0.01	32.1	32.7	7.1
HC8a	0.02	0.02	0.01	12.8	9.8	6.8
HC8a	0.01	0.01	0.01	7.6	4.5	5.0
HC8b	0.01	0.00	0.01	4.4	0.4	9.0
HC8b	0.01	0.01	0.01	4.7	7.2	7.6
HC8b	0.03	0.04	0.04	17.1	25.8	23.1
N1	0.05	0.04	0.04	12.4	11.3	9.9
N3a / N4	0.02	0.06	0.04	3.5	10.2	6.1
N3b	0.04	–	–	–	–	–
N4	0.03	–	–	–	–	–
O1 / S	0.25	0.37	0.20	64.7	96.1	51.6
O2	0.05	0.03	0.01	22.0	13.7	2.9

[a]Energy minimization with B3LYP/aug-cc-pVTZ, PCM = diethylamine.

Table S7: Overview of Deviations in ESP Charges of the Studied Chromophores (TB1, TCT, DNS, DNT)[a]

	Unsigned Absolute Deviation / e			Unsigned Relative Deviation / %		
	TB1−TCT	TB1−DNS	TB1−DNT	TB1−TCT /TB1	TB1−DNS /TB1	TB1−DNT /TB1
Min.	0.0001	0.001	0.0001	0.2	0.4	0.05
Max.	0.52	0.65	0.35	440.6	408.7	861.2
Avg.	0.07	0.07	0.04	34.4	30.2	30.9
H Atoms and Acceptor Group Atoms Excluded						
Min.	0.001	0.004	0.001	0.4	2.4	0.4
Max.	0.34	0.19	0.09	184.8	64.4	43.6
Avg.	0.07	0.04	0.02	40.6	18.0	10.8

[a]Energy minimization with B3LYP/aug-cc-pVTZ, PCM = diethylamine.

of these differences. It is important to note that atomic charge parameters which are not comparable are excluded from further consideration. For instance, N3b of TB1 and N3 of DNT or DNS are not comparable (see Figure S6). The average unsigned relative charge parameter deviation between TB1 and a modified acceptor group chromophore is between 30 % and 34 %. Large maximum deviations arise from hydrogen atoms due to their small partial charges, where minor deviations can lead to substantial relative deviations. Other large maximum deviations arise from atoms in the modified acceptor group or in close proximity to it (compare Table S6 with Figure S6). When hydrogen atoms and acceptor group atoms (all atoms including C7 and beyond C7) are filtered out, the average unsigned relative deviation between the charge parameters of TB1, DNS, and DNT is less than 20 %, and the absolute values differ on average only to the second decimal place. For TCT, the deviations are slightly larger, because mainly the atoms C4 to C6 are more strongly polarized than in DNT and DNS (compare Table S5, Table S6, and Figure S7). Sulfur can delocalize electron density, and when strong acceptors, e.g., nitro groups, are absent, as in TCT, the electron density extends beyond C7 and beyond the acceptor group. Consequently, C5 becomes more negatively charged, while C4 and C6 become more positively charged in the TCT chromophore (compare Table S5, Table S6,

and Figure S7a) and c)). This charge distribution explains the lower dipole moment for TCT in comparison to DNT and DNS.

In order to summarize this subsection, the ESP charges and steric profiles of the different chromophore modifications were examined. It was observed that the steric profile and dipole moment of TB1 changed only slightly. Although larger, more significant deviations in charge parameters are observable in the acceptor group or in atoms in close proximity to it (e.g., in TCT), the overall classical, molecular dynamics phase behavior is expected to be similar for TB1, DNT, TCT, and DNS.

S5 ESP Charge Profiles and Steric Profiles of TB1 in Alternative PCM Environments

This section serves to quantify the uncertainties in the course of polarizable continuum model (PCM) calculations that result from the replacement of solvents not initially provided by the DFT calculation program. In the end of this section, the impact of the environment on the polarizabilities and on the electro-optical effect is assessed. According to our previous study, DEA was used as a substitute for the PMMA environment. To this end, in addition to DEA, we employed 1-iodohexadecane (IHD) and trichloroethene (TCE) as solvents. Table S8 shows the solvent properties utilized in PCM calculations. The

Table S8: Alternative Solvents Applied in PCM Calculations as a Proxy for PMMA, Solvent Properties Adapted from the Minnesota Solvent Descriptor Database[S27,S28]

Environment	ε^a	n^b
Poly(methyl methacrylate) (PMMA)	3.6[c]	1.4785[d]
Diethylamine (DEA)	3.577	1.3864
1-Iodohexadecane (IHD)	3.534	1.4806
Trichloroethene (TCE)	3.422	1.4773

[a]Relative permittivity at 298 K; [b]refractive index at 293 K; [c]ref S29; [d]ref S21,S22.

objective of this investigation was to select solvents that closely resemble the dielectric

environment of PMMA. Consequently, the primary focus was on the relative permittivity ε of the various solvents.

PCM calculations were performed using default settings in Gaussian 16.[S26] The molecular cavity was energy-minimized according to the scaled van der Waals radii of the solute (scale factor: 1.1). Other provided settings include the solvent accessible surface or the solvent excluding surface. The scaled van der Waals radii setting is appropriate, given that the exact molecular cavity between the polymer chains may be highly complex and variable, which is beyond the scope of this study. Furthermore, the objective of this study is to develop a modeling strategy that is straightforward, fast, efficient, accurate, and comprehensible/transparent.

Figure S11 illustrates the steric profiles and the associated dipole moments in different environments. As can be seen in Figure S11, the structural similarity of TB1 in various

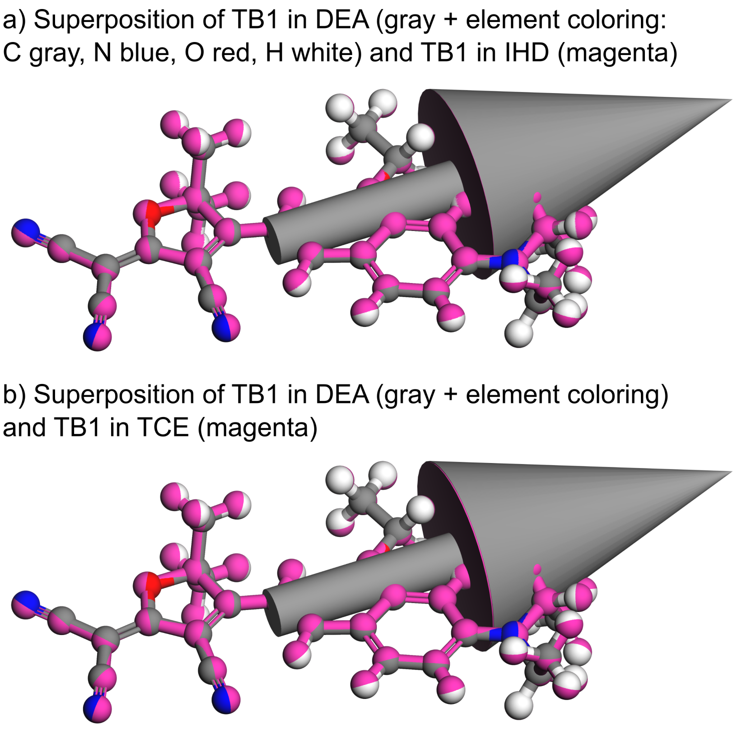

Figure S11: Dipole moments and steric profiles of TB1 in different environments. Results of an energy minimization with B3LYP/aug-cc-pVTZ and PCM = diethylamine (DEA), 1-iodohexadecane (IHD), and trichloroethene (TCE).

solvents is evident, as the different calculated molecular structures align precisely on top of each other. Each atom is associated with two distinct color schemes, representing the maximum number that can be displayed simultaneously.

Figure S12 shows the charge profile of TB1 in the different polarizable environments, introduced in Table S8. The acceptor group is located on the left side and Figure S6 provides a reference presentation with element coloring. The charge profiles do not show any significant differences depending on the solvent. The differences are smaller than those observed for the acceptor group modifications (see Figure S7). The explicit partial charges are discussed in the next paragraph.

Table S9 contains the atomic partial charges for different solvents. A statistical overview of charge deviations is provided at the bottom. On average, the relative unsigned charge deviation is below 0.28 % for TCE and below 0.08 % for IHD compared to DEA. The maximum relative unsigned charge deviation is 6.16 % for TCE and 1.65 % for IHD compared to DEA. As observed in the section on acceptor group modification, the largest charge deviation occurs on hydrogen atoms with a very low charge. Consequently, minor deviations can result in seemingly substantial relative deviations. Due to the minimal average unsigned relative deviations (less than 0.3 %), the variations in the charge parameters are regarded as negligible for the specified solvents. All derived charges appear to correspond well.

Table S10 presents the first absorption wavelength λ_0, the dipole moment μ, the polarizability α and hyperpolarizability β values for TB1 in the different environments. The (hyper)polarizability properties are nearly identical across the different solvation models in the static limit. The first absorption wavelength of TB1 in the DEA model is slightly blue shifted (approx. $\Delta\lambda = -4$ nm) in comparison to the other solvation models. At the wavelength of the electro-optical (EO) experiment ($\lambda = 850$ nm), the polarizabilities and hyperpolarizabilities are nearly identical for IHD and TCE, and slightly smaller for DEA ($\Delta\alpha \approx -2$ % to 3 %; $\Delta\beta = -6$ %). The blue shifted lowest absorption transition and slightly

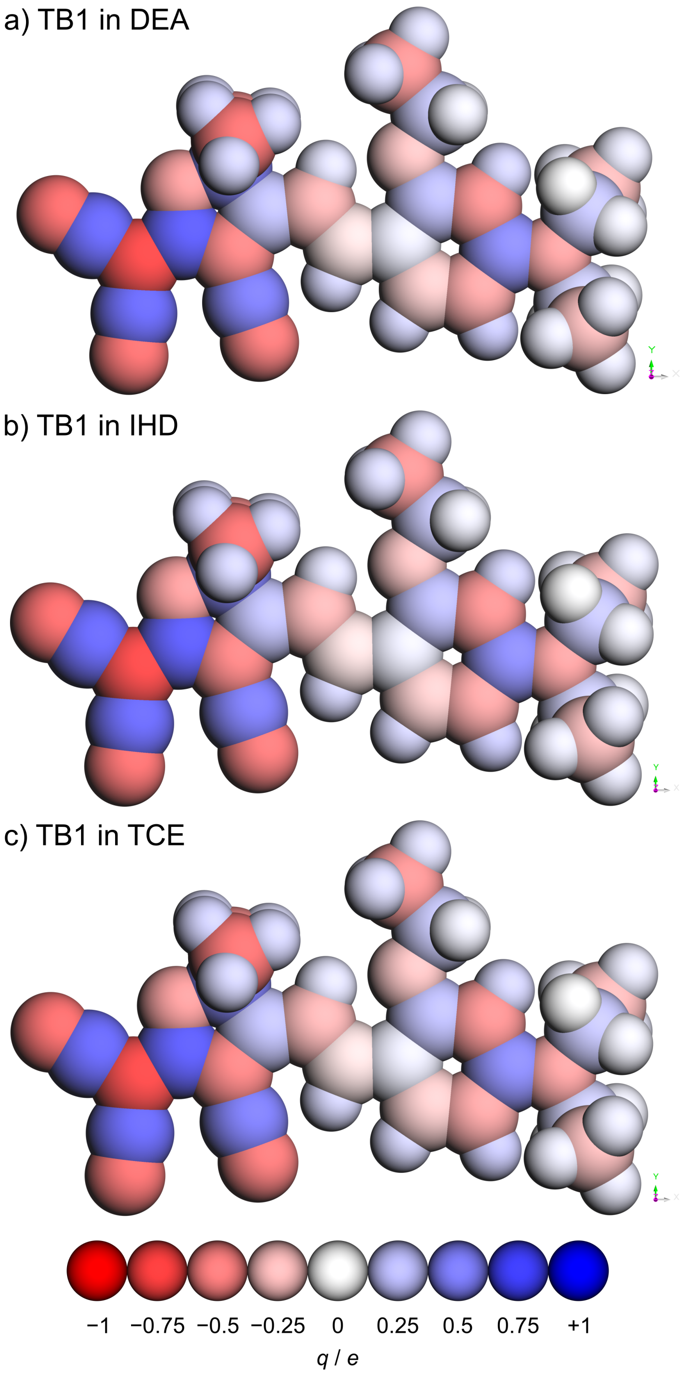

Figure S12: ESP charge profile of TB1 in different environments. Results of an energy minimization with B3LYP/aug-cc-pVTZ, PCM = (a) diethylamine (DEA), (b) 1-iodohexadecane (IHD), and (c) trichloroethene (TCE).

Table S9: ESP Charges of TB1 after Energy Minimization in Different Environments[a]

Atom	Partial Charge of TB1 in ... /e DEA	IHD	TCE	Unsigned Rel. Dev. / % DEA−IHD /DEA	DEA−TCE /DEA
B1	0.21	0.21	0.21	0.01	0.05
B2	−0.42	−0.42	−0.42	0.00	0.00
C1	0.47	0.47	0.47	0.05	0.20
C10	−0.50	−0.50	−0.50	0.07	0.26
C11	0.53	0.53	0.52	0.05	0.18
C12	−0.75	−0.75	−0.75	0.08	0.28
C13a	0.62	0.62	0.61	0.06	0.24
C13b	0.62	0.62	0.62	0.05	0.19
C14a	0.18	0.18	0.18	0.07	0.25
C14b	0.22	0.22	0.22	0.06	0.20
C15a	−0.25	−0.25	−0.25	0.01	0.03
C15b	−0.28	−0.28	−0.28	0.02	0.08
C2a	−0.36	−0.36	−0.36	0.00	0.00
C2b	−0.44	−0.44	−0.44	0.05	0.18
C3a	−0.19	−0.19	−0.19	0.08	0.31
C3b	0.25	0.25	0.25	0.07	0.24
C4	0.07	0.07	0.07	0.03	0.10
C5	−0.11	−0.11	−0.11	0.03	0.12
C6	−0.29	−0.29	−0.29	0.04	0.16
C7	0.24	0.24	0.24	0.12	0.44
C8	0.53	0.53	0.53	0.03	0.13
C8a	−0.49	−0.49	−0.49	0.01	0.04
C8b	−0.56	−0.56	−0.56	0.01	0.03
C9	0.67	0.67	0.67	0.07	0.25
HB1	0.05	0.05	0.05	0.11	0.42
HB1	0.05	0.05	0.05	0.12	0.45
HB2	0.13	0.13	0.13	0.00	0.00
HB2	0.13	0.13	0.13	0.00	0.00
HB2	0.12	0.12	0.12	0.02	0.07
HC14a	0.05	0.05	0.05	0.11	0.42
HC14a	0.01	0.01	0.01	0.02	0.04
HC14b	0.05	0.05	0.05	0.10	0.39
HC14b	0.00	0.00	0.00	1.65	6.16
HC15a	0.07	0.07	0.07	0.04	0.13
HC15a	0.07	0.07	0.07	0.03	0.10
HC15a	0.08	0.08	0.08	0.02	0.09
HC15b	0.08	0.08	0.08	0.01	0.05
HC15b	0.08	0.08	0.08	0.06	0.22
HC15b	0.07	0.07	0.07	0.02	0.07
HC2a	0.20	0.20	0.20	0.04	0.14
HC2b	0.16	0.16	0.16	0.09	0.35
HC3a	0.16	0.16	0.16	0.03	0.12
HC5	0.16	0.16	0.16	0.06	0.21
HC6	0.10	0.10	0.10	0.07	0.27
HC8a	0.14	0.14	0.14	0.08	0.31
HC8a	0.16	0.16	0.16	0.03	0.10
HC8a	0.14	0.14	0.14	0.00	0.01
HC8b	0.16	0.16	0.16	0.00	0.02
HC8b	0.18	0.18	0.18	0.02	0.09
HC8b	0.16	0.16	0.16	0.07	0.28
N1	−0.37	−0.37	−0.37	0.04	0.13
N3a	−0.58	−0.58	−0.58	0.09	0.34
N3b	−0.60	−0.60	−0.60	0.08	0.29
N4	−0.54	−0.54	−0.54	0.08	0.31
O1	−0.39	−0.39	−0.39	0.01	0.05
O2	−0.23	−0.23	−0.23	0.04	0.15
Deviation to DEA		Abs. /e	Abs. /e	Rel. / %	Rel. / %
Min.	–	2.0×10^{-6}	4.0×10^{-6}	7.1×10^{-4}	1.9×10^{-3}
Max.	–	5.8×10^{-4}	2.1×10^{-3}	1.65	6.16
Avg.	–	1.3×10^{-4}	4.8×10^{-4}	0.08	0.28

[a]B3LYP/aug-cc-pVTZ, DEA: diethylamine, IHD: 1-iodohexadecane, TCE: trichloroethene

Table S10: Wavelength of First Absorption λ_0, Dipole Moment μ, Polarizabilities α^a and Hyperpolarizabilities β^b of TB1 in Different Environments^c

TB1 in ...	λ_0 / nm	μ / D	μ / D	α_{xx}	α_{yy}	α_{zz}	$\beta_{\|\|}$	β_{tot}
		B3LYP	CAM-B3LYP	Values for $\lambda = 850$ nm				
DEA	472	27.7	27.0	43.7	64.2	154.3	283.8	473.0
IHD	476	27.6	27.0	44.5	65.7	158.8	302.1	503.5
TCE	475	27.5	26.8	44.5	65.6	158.3	300.2	500.3
				Values for the Static Limit Below				
DEA				45.6	65.3	133.4	179.9	299.8
IHD				45.6	65.2	133.2	179.1	298.5
TCE				45.4	64.9	132.5	177.0	295.1

^{a}in 10^{-24} esu; ^{b}in 10^{-30} esu; ^{c}results of an energy minimization with B3LYP/aug-cc-pVTZ and a subsequent polar calculation with CAM-B3LYP/aug-cc-pVTZ, DEA: diethylamine, IHD: 1-iodohexadecane, TCE: trichloroethene.

lower polarizabilities for the DEA model are attributed to the lower refractive index in comparison to IHD and TCE (compare Table S8).

Table S11 summarizes the effects of the different solvents on the EO effect. As was previously observed for the polarizabilities, the values in the static limit are similar. At the wavelength of the EO experiment, the variations are slightly visible and correspond to the trends in the values of the total hyperpolarizability. The EO activity in DEA is less than 6 % smaller than in IHD.

In summary, the effects of the selected solvents on the charge distribution of the chromophore are marginal or even negligible in this study. The polarizability values in the static limit are barely affected by the type of solvent. However, the lowest absorption wavelength and (hyper)polarizability values in the wavelength range of the EO experiment, may be slightly affected by the refractive index of the selected solvent (compare Table S8). The calculation of the first absorption, in combination with the calculation of polarizability values at several wavelengths in order to obtain the dispersion relation, facilitates the identification of the optimum wavelength range (largest EO effect) for a given chromophore system. A more profound discussion of the uncertainties of (hyper)polarizability values

Table S11: Overview of the Electro-Optic Activity (Values After Relaxation) in Different Environments[a]

Model Set	N_c / 10^{20} cm^{-3}	r_{33} / pm V^{-1}		
		DEA	IHD	TCE
		Values for $\lambda(\text{EO}) = 850$ nm		
6 mol% "TB1"	3.22	14.5 ± 0.9	15.3 ± 1.0	15.2 ± 1.0
8 |	4.07	16.1 ± 1.2	16.9 ± 1.3	16.8 ± 1.2
17 |	6.84	23.7 ± 0.7	24.6 ± 0.8	24.5 ± 0.8
		Values for the Static Limit Below		
6 mol% "TB1"	3.22	9.7 ± 0.6	9.7 ± 0.6	9.6 ± 0.6
8 |	4.07	10.8 ± 0.8	10.8 ± 0.8	10.7 ± 0.8
17 |	6.84	16.5 ± 0.5	16.5 ± 0.5	16.4 ± 0.5

[a]The model set is described by the rounded mole percentage of chromophore MAB1 covalently bonded at the PMMA polymer host. All values are average values of five independent models and their corresponding standard deviations. The model set density ρ and order parameter $\langle\cos^2\theta\rangle$ and $\langle\cos^3\theta\rangle$ are adapted from the MAB1 simulations (see main manuscript, not tabulated here). The errors in number density ΔN_c are all less than 0.007 and are therefore omitted.

in simulation and experiment and the dispersion of the (hyper)polarizability values is presented at the end of section S3. Unless otherwise stated, the PCM model of DEA is selected for all calculations.

References

(S1) Denda, N. M.; Rohloff, E.; Kurth, F. R.; Zhao, L.; Johannes, H.-H.; Kowalsky, W.; König, C.; Behrens, P.; Schneider, A. M. Molecular dynamics simulations of electric field poled poly(methyl methacrylate) doped with tricyanopyrroline chromophores. *J. Phys. Chem. B* **2025**, *129*, 8015–8027.

(S2) Kanis, D. R.; Ratner, M. A.; Marks, T. J. Design and construction of molecular assemblies with large second-order optical nonlinearities. Quantum chemical aspects. *Chem. Rev.* **1994**, *94*, 195–242.

(S3) Johnson, L. E.; Dalton, L. R.; Robinson, B. H. Optimizing calculations of electronic excitations and relative hyperpolarizabilities of electrooptic chromophores. *Acc. Chem. Res.* **2014**, *47*, 3258–3265.

(S4) Iikura, H.; Tsuneda, T.; Yanai, T.; Hirao, K. A long-range correction scheme for generalized-gradient-approximation exchange functionals. *J. Chem. Phys.* **2001**, *115*, 3540–3544.

(S5) Tawada, Y.; Tsuneda, T.; Yanagisawa, S.; Yanai, T.; Hirao, K. A long-range-corrected time-dependent density functional theory. *J. Chem. Phys.* **2004**, *120*, 8425–8433.

(S6) Peverati, R.; Truhlar, D. G. Improving the accuracy of hybrid meta-GGA density functionals by range separation. *J. Phys. Chem. Lett.* **2011**, *2*, 2810–2817.

(S7) Chai, J.-D.; Head-Gordon, M. Systematic optimization of long-range corrected hybrid density functionals. *J. Chem. Phys.* **2008**, *128*, 084106.

(S8) Yanai, T.; Tew, D. P.; Handy, N. C. A new hybrid exchange–correlation functional using the Coulomb-attenuating method (CAM-B3LYP). *Chem. Phys. Lett.* **2004**, *393*, 51–57.

(S9) Becke, A. D. Density-functional thermochemistry. III. The role of exact exchange. *J. Chem. Phys.* **1993**, *98*, 5648–5652.

(S10) Stephens, P. J.; Devlin, F. J.; Chabalowski, C. F.; Frisch, M. J. *Ab initio* calculation of vibrational absorption and circular dichroism spectra using density functional force fields. *J. Phys. Chem.* **1994**, *98*, 11623–11627.

(S11) Dunning, T. H. Gaussian basis sets for use in correlated molecular calculations. I. The atoms boron through neon and hydrogen. *J. Chem. Phys.* **1989**, *90*, 1007–1023.

(S12) Kendall, R. A.; Dunning, T. H.; Harrison, R. J. Electron affinities of the first-row

atoms revisited. Systematic basis sets and wave functions. *J. Chem. Phys.* **1992**, *96*, 6796–6806.

(S13) Davidson, E. R. Comment on "Comment on Dunning's correlation-consistent basis sets". *Chem. Phys. Lett.* **1996**, *260*, 514–518.

(S14) Gerratt, J.; Mills, I. M. Force constants and dipole-moment derivatives of molecules from perturbed Hartree–Fock calculations. I. *J. Chem. Phys.* **1968**, *49*, 1719–1729.

(S15) Dodds, J. L.; McWeeny, R.; Raynes, W. T.; Riley, J. P. SCF theory for multiple perturbations. *Mol. Phys.* **1977**, *33*, 611–617.

(S16) Dodds, J. L.; McWeeny, R.; Sadlej, A. J. Self-consistent perturbation theory. *Mol. Phys.* **1977**, *34*, 1779–1791.

(S17) Miertuš, S.; Scrocco, E.; Tomasi, J. Electrostatic interaction of a solute with a continuum. A direct utilizaion of *ab initio* molecular potentials for the prevision of solvent effects. *Chem. Phys.* **1981**, *55*, 117–129.

(S18) Miertuš, S.; Tomasi, J. Approximate evaluations of the electrostatic free energy and internal energy changes in solution processes. *Chem. Phys.* **1982**, *65*, 239–245.

(S19) Pascual-Ahuir, J. L.; Silla, E.; Tuñon, I. GEPOL: An improved description of molecular surfaces. III. A new algorithm for the computation of a solvent–excluding surface. *J. Comput. Chem.* **1994**, *15*, 1127–1138.

(S20) Paschoal, D.; Dos Santos, H. F. Computational protocol to predict hyperpolarizabilities of large π-conjugated organic push–pull molecules. *Org. Electron.* **2016**, *28*, 111–117.

(S21) Polyanskiy, M. N. Refractiveindex.info database of optical constants. *Sci. Data* **2024**, *11*, 94.

(S22) Zhang, X.; Qiu, J.; Li, X.; Zhao, J.; Liu, L. Complex refractive indices measurements of polymers in visible and near-infrared bands. *Appl. Opt.* **2020**, *59*, 2337–2344.

(S23) Besler, B. H.; Merz, K. M.; Kollman, P. A. Atomic charges derived from semiempirical methods. *J. Comput. Chem.* **1990**, *11*, 431–439.

(S24) Singh, U. C.; Kollman, P. A. An approach to computing electrostatic charges for molecules. *J. Comput. Chem.* **1984**, *5*, 129–145.

(S25) BIOVIA Dassault Systèmes Materials Studio 2023, Version: 23.1. 2023.

(S26) Frisch, M. J.; Trucks, G. W.; Schlegel, H. B.; Scuseria, G. E.; Robb, M. A.; Cheeseman, J. R.; Scalmani, G.; Barone, V.; Petersson, G. A.; Nakatsuji, H.; Li, X.; Caricato, M.; Marenich, A. V.; Bloino, J.; Janesko, B. G.; Gomperts, R.; Mennucci, B.; Hratchian, H. P.; Ortiz, J. V.; Izmaylov, A. F.; Sonnenberg, J. L.; Williams-Young, D.; Ding, F.; Lipparini, F.; Egidi, F.; Goings, J.; Peng, B.; Petrone, A.; Henderson, T.; Ranasinghe, D.; Zakrzewski, V. G.; Gao, J.; Rega, N.; Zheng, G.; Liang, W.; Hada, M.; Ehara, M.; Toyota, K.; Fukuda, R.; Hasegawa, J.; Ishida, M.; Nakajima, T.; Honda, Y.; Kitao, O.; Nakai, H.; Vreven, T.; Throssell, K.; Montgomery, J. A., Jr.; Peralta, J. E.; Ogliaro, F.; Bearpark, M. J.; Heyd, J. J.; Brothers, E. N.; Kudin, K. N.; Staroverov, V. N.; Keith, T. A.; Kobayashi, R.; Normand, J.; Raghavachari, K.; Rendell, A. P.; Burant, J. C.; Iyengar, S. S.; Tomasi, J.; Cossi, M.; Millam, J. M.; Klene, M.; Adamo, C.; Cammi, R.; Ochterski, J. W.; Martin, R. L.; Morokuma, K.; Farkas, O.; Foresman, J. B.; Fox, D. J. Gaussian 16, revision B.01. 2016.

(S27) Marenich, A. V.; Cramer, C. J.; Truhlar, D. G. Universal solvation model based on solute electron density and on a continuum model of the solvent defined by the bulk dielectric constant and atomic surface tensions. *J. Phys. Chem. B* **2009**, *113*, 6378–6396.

(S28) Marenich, A. V.; Kelly, C. P.; Thompson, J. D.; Hawkins, G. D.; Chambers, C. C.;

Giesen, D. J.; Winget, P.; Cramer, C. J.; Truhlar, D. G. Minnesota Solvation Database (MNSOL) version 2012.

(S29) Brandrup, J.; Immergut, E. H.; Grulke, E. A.; Abe, A.; Bloch, D. R. *Polymer Handbook*, 4th ed.; A Wiley-Interscience Publication; Wiley: New York and Weinheim, 1999.
